# Comparing YOLOv3, YOLOv4 and YOLOv5 for Autonomous Landing Spot Detection in Faulty UAVs

**DOI:** 10.3390/s22020464

**Published:** 2022-01-08

**Authors:** Upesh Nepal, Hossein Eslamiat

**Affiliations:** Mechanical, Aerospace, and Material Engineering, Southern Illinois University Carbondale, 1230 Lincoln Dr, Carbondale, IL 62901, USA; upesh.nepal@siu.edu

**Keywords:** object detection, DOTA aerial image dataset, deep learning, YOLOv3, YOLOv4, YOLOv5, unmanned aerial vehicle, UAV Safety, neural networks

## Abstract

In-flight system failure is one of the major safety concerns in the operation of unmanned aerial vehicles (UAVs) in urban environments. To address this concern, a safety framework consisting of following three main tasks can be utilized: (1) Monitoring health of the UAV and detecting failures, (2) Finding potential safe landing spots in case a critical failure is detected in step 1, and (3) Steering the UAV to a safe landing spot found in step 2. In this paper, we specifically look at the second task, where we investigate the feasibility of utilizing object detection methods to spot safe landing spots in case the UAV suffers an in-flight failure. Particularly, we investigate different versions of the YOLO objection detection method and compare their performances for the specific application of detecting a safe landing location for a UAV that has suffered an in-flight failure. We compare the performance of YOLOv3, YOLOv4, and YOLOv5l while training them by a large aerial image dataset called DOTA in a Personal Computer (PC) and also a Companion Computer (CC). We plan to use the chosen algorithm on a CC that can be attached to a UAV, and the PC is used to verify the trends that we see between the algorithms on the CC. We confirm the feasibility of utilizing these algorithms for effective emergency landing spot detection and report their accuracy and speed for that specific application. Our investigation also shows that the YOLOv5l algorithm outperforms YOLOv4 and YOLOv3 in terms of accuracy of detection while maintaining a slightly slower inference speed.

## 1. Introduction and Related Works

UAVs are extensively being used in many fields, such as traffic monitoring, surveillance, inspection, surveys, etc. They have replaced choppers in recent years due to their higher mobility and flexibility [1]. The advancement of real-time deep learning algorithms with improved speed and accuracy is changing how UAVs are being utilized in modern society. Recently, UAVs have come to dominate aerial sensing research with the use of deep neural networks in the urban, environmental, and agricultural contexts [2].

In this work, we address a safety concern related to using UAVs in urban environments. We choose urban environments since the risk of catastrophic accidents because of faulty UAVs is higher there, due to higher infrastructures and population density compared to rural and other environments. So, if we address the problem in urban environments then the problem in other environments will be automatically addressed. In other words, in urban environments safe and uncluttered landing zones are harder to find compared to other environments. Therefore, addressing the safety concern in urban environments is more challenging compared to other environments. Hence, we tackle the problem in urban environments. In other environments, e.g., rural areas, uncluttered zones are easier to find, and if the safety system can work well for a city, it can also work for rural areas Specifically, we consider a UAV that has suffered an in-flight system failure and needs to make an emergency landing. This operation can be divided into three main tasks. The first task is to detect that there is a failure. This can be done by monitoring health of the UAV continuously, and using a clustering method, such as K-Means clustering similar to [3], to classify different health states. Then, if a failure is detected, in the second task the UAV needs to find a safe landing spot to perform an emergency landing. In the third and final step, the UAV needs to be steered to reach the selected landing spot. In this paper, we are interested in the second step, i.e., selecting a safe landing spot for emergency landing. It is worth noting that once a failure is detected, the chosen YOLO algorithm starts and when it finds a potential landing spot, it can stop, and then the third step of the framework is triggered. In other words, the landing spot detection algorithm does *not* need to identify different landing spots continuously, rather, it should find (at least) one landing spot quickly, and then it can stop. From the safety perspective, half a second is considered quick enough for finding a safe landing spot, so landing spot detection algorithms with a minimum FPS of 2 satisfy the requirement for safety. The other important factor in choosing the best algorithm for emergency landing spot detection is accuracy. The best algorithm will have the highest accuracy in emergency landing spot detection, while ensuring a quick detection speed that satisfies the safety requirement.

Currently, most of UAV flights are operated by a remote human pilot. However, this has been changed by advances in autonomous UAVs, whereby an autonomous flight controller makes most of the decisions, instead of a human pilot. Autonomous UAVs have the capability to outperform human pilots because they can make better and faster decisions, while considering many more factors. Additionally, pilots are expensive to train, may not always be available and are also prone to errors. The safety measure that we propose in this paper can help both autonomous UAVs and human-operated UAVs. In the former, the emergency landing spot detection system selects a safe landing spot, and then the UAV is autonomously steered to that landing spot, which is achieved by the third task of this operation. In case that the UAV is operated by a human pilot, the system will suggest the best emergency landing spot to the pilot. It is the pilot who will then steer the vehicle to that landing spot.

Here, we investigate the feasibility of using three state-of-the-art object detection algorithms in task two. Specifically, we compare the accuracy and speed of YOLOv3, YOLOv4 and YOLOv5l [4,5,6,7] for the purpose of emergency landing spot detection using aerial images. We use the DOTA dataset [8,9,10] to train and evaluate these models. The DOTA dataset is a collection of 11268 satellite and aerial images taken from Google Earth, GF-2, and JL-1 satellites with pixel range of 800 × 800 to 20,000 × 20,000. We divide the dataset into training and test set in the ratio 3:1. Images are classified into 15 classes of plane, ship, storage tank, baseball diamond, tennis court, basketball court, ground track field, harbor, bridge, large vehicle, small vehicle, helicopter, roundabout, soccer ball field and swimming pool. We categorize the baseball diamond, tennis court, basketball court, ground track field, soccer ball field, roundabout, and swimming pool as admissible places to land and plane, ship, storage tank, harbor, bridge, large vehicle, small vehicle, and the helicopter as locations to avoid. The DOTA dataset has been carefully selected because it meets the requirements of our application, i.e., it has the objects that we expect a UAV to see in a rural flight, from realistic heights and angles. DOTA dataset has also sufficient samples and does not require additional images for training and validation for our specific application of emergency landing spot detection. We expect the results to hold true for other datasets as long as the objects in those datasets are seen with similar angles and heights to those of DOTA’s. During the training of neural networks, images with large sizes require very high GPU capacity. To solve this problem, we split the large images into smaller elements for training. Therefore, we were able to use the GPU mentioned in the training the algorithm section.

**Figure 1:**
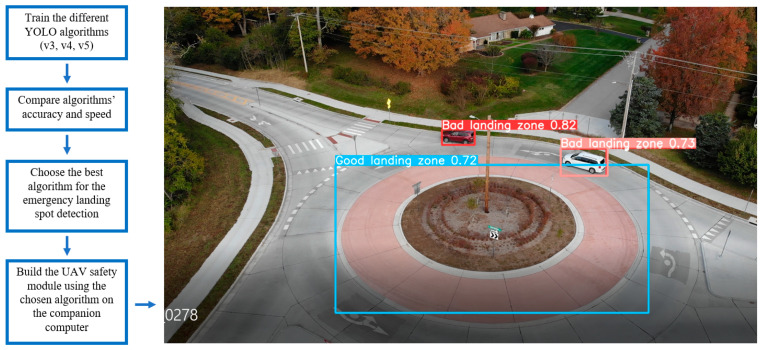
Flowchart of the steps involved in this work (**left**) and the output of the chosen algorithm (YOLOv5l) working on a video stream of a UAV flying near Southern Illinois University campus (**right**) All UAV flights for this project were operated in accordance with FAA regulations.

There are peer reviewed comparisons of YOLOv3 with SSD (Single Shot multi-box detector) [11], Faster R-CNN [12], and other real-time deep learning algorithms. However, YOLOv3, YOLOv4 and YOLOv5 are yet to be compared for our application in the aerial image object detection. Comparisons have been made between YOLOv3, YOLOv4, and YOLOv5, in which some authors claim that YOLOv4 is efficient [13] while others claim that YOLOv5 is efficient [14]. To bridge this gap, we compare those three algorithms using DOTA [8,9,10], without changing any hyperparameters. We use mAP and F1 scores [15] to measure the accuracy and utilize FPS (Frames Per Second) to compare speed of the three YOLO algorithms. We compare the speed both in a PC and a CC, and eventually plan to use the best algorithm on the CC attached to a UAV. We are comparing the algorithms in the PC for verification purposes; we expect to see a similar trend of speed and accuracy between algorithms in the PC and the CC, so that we can verify the results that we get from the CC. Therefore, we compare the speed and accuracy both in the PC and the CC. The steps involved in this work are shown in Figure 1.

Deep learning has been applied to many different fields, from forecasting water quality [16] to autonomous trajectory generation for UAVs [17,18]. Due to the advancements in deep learning technology, object classification speed has achieved impressive milestones. There has also been a significant improvement in accuracy of these algorithms in recent years. We can have real-time object detection with these algorithms, which makes it suitable for use in robotics and UAV applications. The use of Graphics Processing Units (GPUs) in deep learning algorithms has also contributed to the evolution of computer vision and deep learning techniques [19]. This enables us to use object detection algorithms suitable in development of real-time applications.

Since 2012, various CNN [20] algorithms are proposed. R-CNN and its variants use region proposal method [21,22,23]. Instead of running the detection in a single image, these methods first divide each image into different regions using selective search method [21] and support vector machine (SVM) [24] is used to classify those regions into classes. This process requires a lot of time to train and make predictions. To overcome the limitations of R-CNN, Fast R-CNN [21] is proposed, which uses convolution feature map of an image as an input instead of the region method used by R-CNN. In another study [25] an update in Fast-RCNN has been proposed, called Faster R-CNN, which replaces selective search with Region Proposal Network. On the other hand, YOLO, proposed in 2016, uses a single image as the input without dividing the image in regions. This makes detection speed much faster.

We continue this section by presenting the comparison of YOLO algorithm with other state-of-the-art real-time deep learning algorithms in the related literature. In [12], ‘Faster R-CNN’ and YOLOv3 are compared for object detection. This study uses a custom-made dataset of 218 training images and 52 test images. This study concluded that YOLOv3 outperforms Faster R-CNN in terms of both speed and accuracy. 

In [26], Faster R-CNN, YOLOv3 and SSD are compared for object detection using Remote sensing images collected from GF-1 and GF-2 satellites. It uses a training dataset of 826 images and a testing dataset of 275 images. This study concluded that YOLOv3 has higher mAP and FPS than SSD and Faster R-CNN models.

In [27], Faster R-CNN, SSD, YOLOv3 are compared for object detection using Google earth images and DOTA dataset. It uses 224 images for training and 56 images for testing purposes with a resolution from 600 × 600 to 1500 × 1500. This study concluded that YOLOv3 has higher mAP and FPS than Faster R-CNN and SSD.

In [28], Mask R-CNN architecture is compared with YOLOv3. The dataset contains 800 training and 70 test images It was found that the accuracy of Mask R-CNN is significantly higher compared to YOLOv3, but in terms of detection speed, YOLOv3 outperformed Mask R-CNN. Specifically, the detection speed of YOLOv3 was 3 times higher compared to that of Mask R-CNN. 

In [29], YOLOv4 is compared with SSD and Faster R-CNN. The dataset consists of 2620 training and 568 test images. It was found that the accuracy of YOLOv4 is significantly higher compared to SSD and Faster R-CNN whereas, the detection speed of SSD is higher compared to YOLOv3 and Faster R-CNN. The performance of Faster R-CNN is poor both in terms of accuracy and speed. 

In [13], YOLOv4 is compared with YOLO5. The dataset contains 5939 images of both faulty and normal pin and disk insulator images for training and 1400 test images. It was found that the accuracy of YOLOv4 is higher compared to the accuracy of YOLOv5. 

In [30], YOLOv3 is compared with YOLOv4. MS COCO dataset was used in the training and testing the algorithms. It was found that YOLOv4 outperformed YOLv3 in terms of accuracy in MS COCO dataset.

In [7], YOLOv3 is compared with YOLOv4. MS COCO dataset was used in the training and testing the algorithm. It was found that YOLOv4 outperformed YOLOv3 in terms of accuracy and detection speed.

In [14], YOLOv3, YOLOv4, and YOLOv5 are compared. MS COCO dataset is used in training and testing the algorithms. It was found that YOLOv5 outperforms YOLOv4 and YOLOv3 in terms of accuracy. The detection speed of YOLOv3 was faster compared to YOLOv4 and YOLOv5 and the detection speed of YOLOv4 and YOLOv5 were identical.

In this paper, we consider YOLOv3, YOLOv4, and YOLOv5l for comparison. These are state-of-the-art real-time deep learning algorithms used for object detection. We selected these algorithms because of their high performance in real-time applications, based on the aforementioned related work in the previous paragraph. Table 1 summarizes related comparison of real-time deep learning algorithms from literature review. We found that YOLOv3 is accurate and faster compared to other deep learning algorithms such as Faster R-CNN and SSD. From the literature we also see that YOLOv4 is more accurate compared to YOLOv3; however, the reported accuracy of YOLOv4 versus YOLOv5 is still open to question as some authors claim that YOLOv4 is more accurate while others claim that YOLOv5 is more accurate. The reason for different reported results can be attributed to many factors, such as the different datasets used, modified hyperparameters, etc. These differences are stemming from particular applications that other researchers have looked at. Since none of the related works use aerial images while comparing different YOLO algorithms with the specific criterion for our safety system, we conduct a comparative study for emergency landing spot detection to bridge that gap. The contributions of this paper are as follows: First, we consider three different YOLO algorithms for emergency landing spot detection problem to investigate their impact on the performance of the proposed safety framework. In addition, we verify the differences in their performance trends on a CC with a PC. Finally, we confirm the feasibility of using such algorithms for utilization in the safety framework that requires the algorithm to run quick enough on a CC, while being accurate.

The rest of this paper is organized as follows: In Section 2, we discuss the theoretical overview and architecture of YOLO algorithms. In Section 3, evaluation metrics are discussed, followed by training and comparison methods and results. We conclude the paper in Section 4 followed by Appendix A.

## 2. Theoretical Overview

Deep learning algorithms fall under the following two categories: single-stage classifiers and two-stage classifiers. Two-stage classifiers generate regions which may contain objects. These regions are then classified into objects by a neural network. Therefore, they are generally more accurate than single-stage classifiers, however, they have slower inference speed because of the multiple stages involved in the detection process. On the other hand, in single-stage detectors, region proposal step is removed and both object localization and classification are done in the same step. This makes single-stage classifiers faster compared to multiple-stage classifiers.

YOLO is a single stage deep learning algorithm which uses convolution neural network for object detection. It is popular due to its speed and accuracy. There are various deep learning algorithms, but they are unable detect an object in a single run but YOLO, on the other hand, makes the detection in a single forward propagation through a neural network which makes it suitable for real-time application. This property has made YOLO algorithm popular among the other deep learning algorithms. 

**Figure 2:**
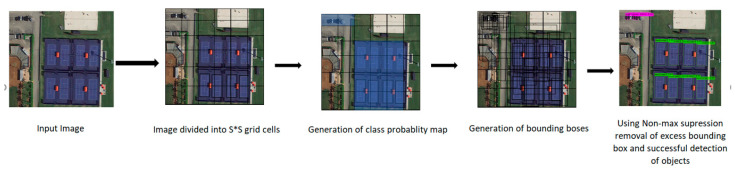
General Architecture of YOLO algorithm.

YOLOv1 divides image into S × S grid cells of equal dimensions. Each grid cell is responsible for object detection if the center of the objects falls inside the cell. Each cell can predict fixed B number of bounding boxes with a confidence score. Each bounding box is composed of 5 values of x, y, w, h, and confidence score. Here, x, y, w, and h are at the center of the bounding box, width, and height, respectively. After the prediction of a bounding box, YOLO uses IOU (Intersection Over Union) to choose right bounding box of an object for the grid cell. To remove excess bounding boxes YOLO uses non-max suppression. If IOU ≥ 0.5 then non-max suppression removes the excess bounding boxes with low confidence score. To calculate loss, YOLO uses the sum of squared error. In YOLOv2 batch normalization was added together with convolution layers to improve the accuracy and reduce the overfitting problem [6]. In YOLOv3, feature extraction backbone of Darknet19 [31], which struggled in detecting small objects, was changed to Darknet 53 to address this problem. In that work, Residual block, skip connections and up-sampling were introduced, which significantly improved the accuracy of the algorithm. In YOLOv4 again the feature extractors backbone was changed to CSPDarknet53, that significantly improved the speed and accuracy of the algorithm. YOLOv5 is the latest and the lightweight version of previous YOLO algorithms and uses PyTorch framework instead of Darknet framework. Figure 2 shows the general architecture of the YOLO algorithm, and Table 2 summarizes the comparison between YOLOv3, YOLOv4 and YOLOv5 algorithm architectures. The head and neural network type are the same for all of the algorithms, whereas backbone, neck, and loss function are different.

In YOLOv3, Darknet53 is used as the backbone to extract features from an input image. Backbone of a deep neural network is composed of convolution layer whose function is to extract essential features from the input image. It uses feature pyramid network (FPN) [32] as a neck. The neck plays an important role to extract features maps from different stages which is composed of several bottom-up and top-down paths and the head is composed of YOLO layer. The role of head in one stage detector is to perform final prediction which is composed of a vector containing bounding box coordinates: width, height, class label, and class probability. First, the image is fed to Darknet53 for feature extraction and afterwards fed to feature pyramid network for feature fusion. Finally, YOLO layer generates the results.

### 2.1. YOLOv4 Architecture

As a modified version of YOLOv3, YOLO4. uses Cross Stage Partial Network (CSPNet) in Darknet, creating a new feature extractor backbone called CSPDarknet53. The convolution architecture is based on modified DenseNet [33]. It transfers a copy of feature map from the base layer to the next layer through dense block. The advantages of using DenseNet include the diminishing gradient vanishing problems, boosting backpropagation, removal of the computational bottleneck, and improved learning. Neck is composed of spatial pyramid pooling (SPP) layer and PANet path aggregation. SPP layer and PANet path aggregation are used for feature aggregation to improve the receptive field and short out important features from the backbone. In addition, the head is composed of YOLO layer. First, the image is fed to CSPDarknet53 for feature extraction and then fed to path aggregation network PANet for fusion. Finally, YOLO layer generates the results, similar to YOLOv3 YOLOv4 uses bag of freebies [34] and bag of specials [7] to improve the algorithm performance. Bag of freebies includes Complete IOU loss (CIOU), drop block regularization and different augmentation techniques. Bags of specials includes mish activation, Diou-NMS [35] and modified the path aggregation networks.

### 2.2. YOLOv5 Architecture

**Figure 3:**
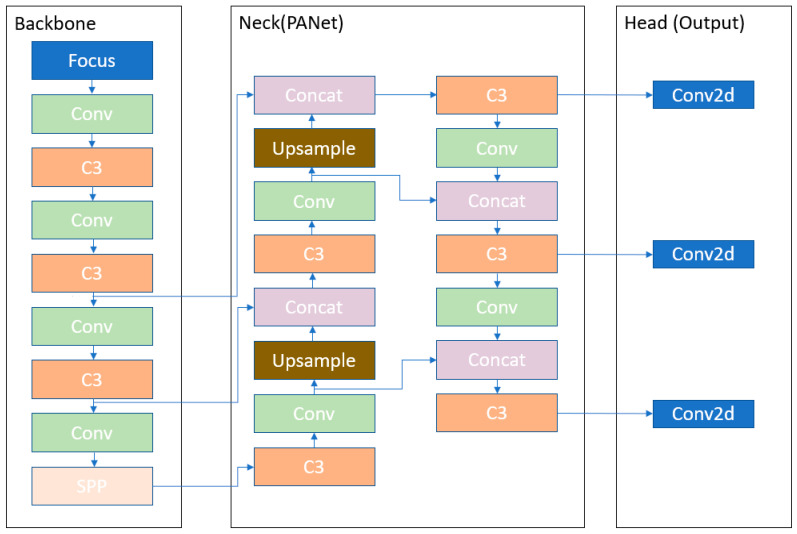
YOLOv5 Architecture.

However, YOLOv5 is different from the previous releases. It utilizes PyTorch instead of Darknet. It utilizes CSPDarknet53 as backbone. This backbone solves the repetitive gradient information in large backbones and integrates gradient change into feature map that reduces the inference speed, increases accuracy, and reduces the model size by decreasing the parameters. It uses path aggregation network (PANet) as neck to boost the information flow. PANet adopts a new feature pyramid network (FPN) that includes several bottom ups and top down layers. This improves the propagation of low level features in the model. PANet improves the localization in lower layers, which enhances the localization accuracy of the object. In addition, the head in YOLOv5 is the same as YOLOv4 and YOLOv3 which generates three different output of feature maps to achieve multi scale prediction. It also helps to enhance the prediction of small to large objects efficiently in the model. The image is fed to CSPDarknet53 for feature extraction and again fed to PANet for feature fusion. Finally, the YOLO layer generates the results. In Figure 3 the architecture of YOLOv5l algorithm is presented. The Focus layer [36] is evolved from YOLOv3 structure. It replaces the first three layers of YOLOv3 and create a single layer in YOLOv5. Additionally, here Conv denotes a convolution layer. C3 is composed of three convolution layers and a module cascaded by various bottlenecks. Spatial pyramid pooling (SPP) is a pooling layer that is used to remove the fixed size constraint of the network. Upsample is used in upsampling the previous layer fusion in the nearest node. Concat is a slicing layer and is used to slice the previous layer. The last 3 Conv2d are detection modules used in the head of the network.

The main differences between YOLOv3, YOLOv4, and YOLOv5 architecture is that YOLOv3 uses Darknet53 backbone. YOLOv4 architecture uses CSPdarknet53 as a backbone and YOLOv5 uses Focus structure with CSPdarknet53 as a backbone. The Focus layer is first introduced in YOLOv5. The Focus layer replaces the first three layers in the YOLOv3 algorithm. The advantage of using a Focus layer is reduced required CUDA memory, reduced layer, increased forward propagation, and backpropagation [36].

## 3. Results and Discussion

### 3.1. Evaluation Metrics

We use F1 score and mAP [15] as the criteria to compare the YOLOv3, YOLOv4, and YOLOv5l algorithms. F1 score is the harmonic mean of precision and recall [37], shown in Equation (2). It is also the model’s test accuracy. The highest possible value of F1 score is 1, which indicates perfect precision and recall, and the lowest possible score is 0, which indicates either the precision or recall is zero. In addition, mAP is calculated by taking mean of average precision (AP) of all the classes, as shown in Equation (1), where q is the number of queries and AveP(q) is the average precision for that given query. Then, mAP can be calculated by taking the mean of AP. mAP can also be considered a measure to calculate the accuracy of machine learning algorithms. In the emergency landing spot detection problem, the True Positive is the number of good (safe and uncluttered) landing spots detected by the algorithm. The False positive is the number non-good landing spots falsely detected by the algorithm as good landing spots, and false negative is the number of good landing spots missed by the algorithm. In addition, we use FPS to evaluate the inference speed of algorithms. FPS is inversely proportional to the time taken to process a single frame of the video. Additionally, it is worth noting that we use the Intersection over union (IOU) threshold to calculate precision and recall. IOU is the ratio between area of overlap and area of union of the ground truth label and the prediction label. Specifically, IOU threshold is used to classify whether the prediction is true positive or false positive. After calculating precision and recall for different IOU thresholds, precision and recall plot is created for a single classifier at different IOU thresholds. Then, the average precision is calculated from the precision-recall curve. As mentioned before, mAP is calculated by taking the mean of average precision (AP) of all the classes. 

Note that precision is calculated as the ratio of true prediction to the total number of predictions. For example, if a model makes 50 predictions and all of them are correct, the precision is 100 percent. Precision does not consider the actual number of true objects present in an image; however, recall calculates the ratio of true predictions to the total number of objects present in an image. For example, if a model detects 75 true objects and there are 100 true objects in the image, then recall is calculated to be 75 percent. Having only high precision or only high recall does not necessarily mean the model is accurate. There should be a balance between both precision and recall in order for an object detection algorithm to be considered accurate. Therefore, we look at the F1 score to decide whether a model is accurate or not.

Our goal is to find an algorithm that can be used on a CC for real-time applications, specifically for emergency landing spot detection of UAVs that have suffered a system failure. Since the algorithm needs to detect good landing spots quickly, FPS also plays an important role here.

(1) $$mAP=\overset{Q}{\underset{q=1}{\sum }}\frac{AveP\left(q\right)}{Q},$$

(2) $$F1score=2*\frac{\left(precision*recall\right)}{precision+recall},$$

(3) $$Precision=\frac{TruePositive}{TruePositive+FalsePositive},$$

(4) $$Recall=\frac{TruePositive}{TruePositive+FalseNegative},$$

### 3.2. Training and Comparing the Algorithms

For training the neural network, we first used YOLOv3 with stochastic gradient descent as a training optimizer with the momentum set to 0.9. The learning rate and weight decay are set to 0.001 and 0.0005, respectively. Height and width of the training images are 416 and 416, respectively.

Similarly, we used YOLOv4 and YOLOv5l for training, with the exact same parameter assignment that we used for YOLOv3. Table 3 shows the comparison results of the three different YOLO algorithms trained using the DOTA dataset for emergency landing spot detection in aerial images. YOLOv5l presents higher mAP and F1 score compared to YOLOv3 and YOLOv4, and that shows the YOLOv5l can detect objects more accurately compared to the other two algorithms for our specific application in the DOTA dataset. In this study, we also see that YOLOv3 is faster than YOLOv4 and YOLOv5l. The higher accuracy of YOLOv5l compared to YOLOv4 is because YOLOv5l uses auto learning bounding boxes [38] which improves the overall accuracy of the algorithm. The higher accuracy of YOLOv4 and YOLOv5l compared to YOLOv3 is due to YOLOv3 using Darknet53 which struggles in detecting small objects whereas YOLOv4 and YOLOv5l use CSPdarkent53 that increases the accuracy significantly. Moreover, YOLOv4 and YOLOv5l use bag of freebies [34], bag of specials [7] and mosaic data augmentation [39] which also increase the accuracy of YOLOv4 algorithm. 

**Figure 4:**
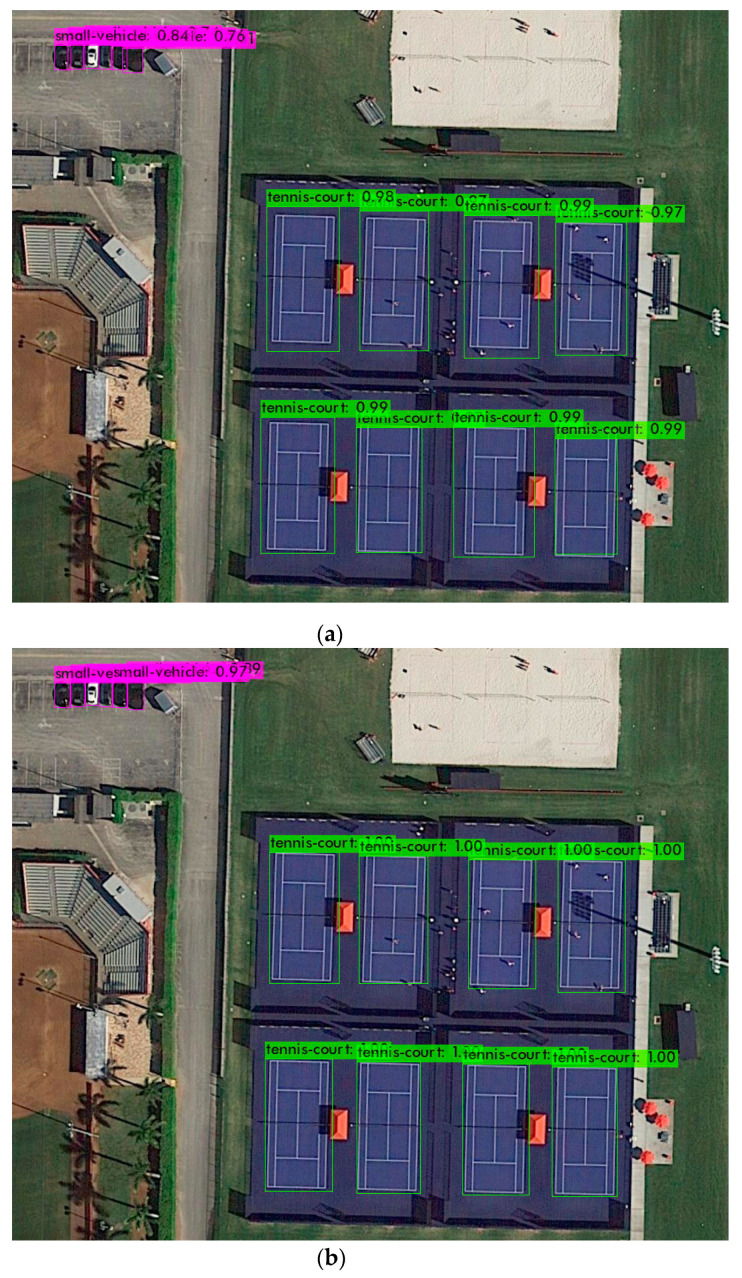

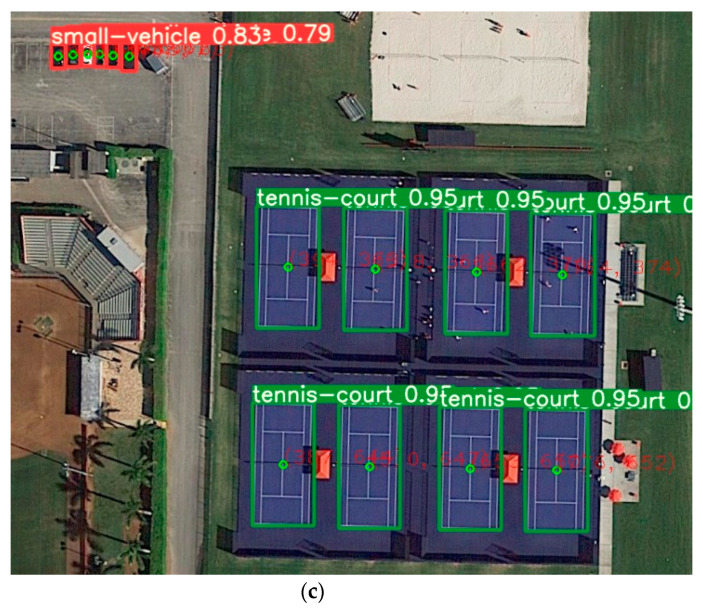
Object detection using YOLO algorithms: (**a**) Object detection using YOLOv3; (**b**) Object detection using YOLOv4; (**c**) Object detection using YOLOv5l.

**Figure 5:**
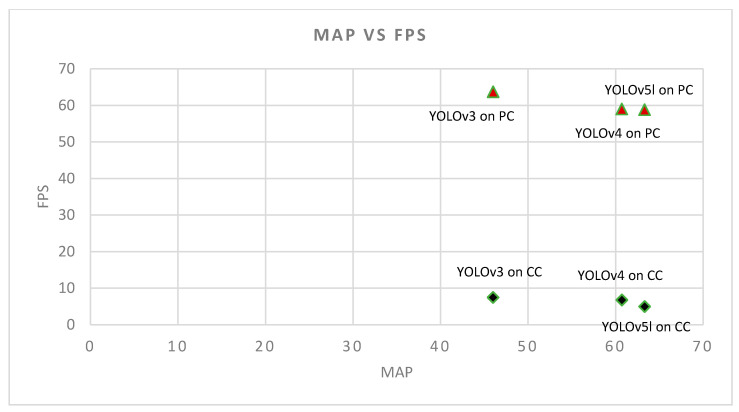
Performance of YOLOv3, YOLOv4, and YOLOv5l in PC (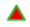) and Companion Computer (CC) (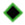). This figure shows that the accuracy of YOLOv5l is higher than YOLOv4 and YOLOv3 with a negligible drop in speed compared to YOLOv4 and YOLOv3.

Figure 4 shows the output of the YOLO algorithms when applied to a sample image. For more images and a video please see the Supplementary Material at the end of Section 4. In addition, the performance of YOLO algorithms in both PC and CC are shown in Figure 5. Detailed results are also presented in Table A1, which shows average precision results of the three YOLO algorithms for all the labels. In addition, Table 3 shows the precision and recall of those algorithms; YOLOv3 has a high precision but its recall is low, and that shows the model needs improvement. For an algorithm to be considered efficient in our work, there must be a balance between precision and recall and that is reflected by the F1 score of the algorithm. As we can see in YOLOv4 and YOLOv5l, their precision and recall are balanced. Therefore, the F1 score of YOLOv4 and YOLOv5l are higher compared to YOLOv3, although YOLOv3 has higher precision. We see that the models in YOLOv4 and YOLv5 have balanced precision and recall which results in a high F1 score.

We use a PC and a CC with the following specifications:

PC Specification:

- CPU: 10th Gen. Intel^®^ Core™ i7-10750H Processor.
- GPU: NVIDIA^®^ GeForce^®^ RTX 2070 SUPER™ Turing™ architecture with 8 GB GDDR6.
- RAM: 32 GB DDR4.
- Storage: 1TB NVMe SSD.
- Operating System: UBUNTU 18.04.

CC Specification:

- Model: Jetson Xavier NX.
- CPU: 6-core NVIDIA Carmel ARM^®^v8.2 64-bit CPU 6MB L2 + 4 MB L3.
- GPU: NVIDIA Volta™ architecture with 384 NVIDIA^®^CUDA^®^ cores and 48 Tenor cores.
- RAM: 8 GB 128-bit LPDDR4x 59.7 GB/s.
- Storage: 500 GB NVMe SSD and.
- Operating System: linux4tegra operating system (an Ubuntu derived OS).

We also used Google Colab with Tesla P100-PCIE-16GB graphics cards to train the neural networks. It provides free and paid access to Google cloud computing resources that can be used in different computing applications. We see that YOLOv4 and YOLOv5l perform better than YOLOv3 in terms of accuracy. We train YOLOv5l in the PyTorch framework, and YOLOv3 and YOLOv4 in the Darknet framework. We use a different framework for YOLOv5l because YOLOv4 and YOLOv3 are developed in the Darknet framework whereas YOLOv5l is developed in PyTorch framework. 

### 3.3. Embedded Platform Results

We are building a standalone module that runs the deep-learning algorithm to detect good and bad landing spots for a faulty UAV. Therefore, we choose to compare the speed of YOLOv3, YOLOv4, and YOLOv5l in a CC. We choose the Nvidia Jetson Xavier NX module in this experiment because of its light weight, energy efficiency, and compact design. YOLOv3 achieved 7.5 FPS, YOLOv4 achieved 6.8 FPS, and YOLOv5l achieved 5 FPS. This shows that these algorithms can be used in real time for landing spot detection with Jetson Xavier NX. We observe that YOLOv3 is faster compared to YOLOv4 and YOLOv5l.YOLOv5l. We also see that YOLOv4′s speed is faster compared to YOLOv5l but slower compared to YOLOv3. Note that the speed of the detection is directly associated with the hardware of the system, and the PC has a better computational capability compared to the CC. Since hardware configurations of the PC and CC are different, we do not expect to see a similar difference between algorithms on the PC and the CC. Hence, if on the CC the YOLOv3 is faster than YOLOv5l with a factor of 1.5, we do not expect to see the same factor on the PC between them. Rather, we see a relatively narrow range of FPS in both PC and CC; FPS range in PC is (63.7 − 58.82 = 4.88) and FPS range in CC is (7.5 − 5 = 2.5).

From Table 3 and Figure 5 we can observe that all three algorithms satisfy the safety requirement as discussed in the Introduction; to have a speed minimum of 2 FPS on a CC. The other important factor in choosing the best algorithm for emergency landing spot detection is accuracy. Therefore, based on Table 3 and Figure 5 we can choose the algorithm with the highest accuracy, and that is YOLOv5l. It has the highest accuracy in emergency landing spot detection, while ensuring quick detection speed that satisfies the safety requirement. Additionally, note that we are using a PC for verification purposes only; we expect to see a similar trend between the three algorithms in a PC and in a CC. Our investigations confirm that; YOLOv5l has the highest mAP compared to YOLOv4 and YOLOv3 on a CC, and the comparisons done in the PC verifies that. Additionally, using YOLOv5l results in a slight drop in speed (−2.5 FPS) compared to YOLOv3, on a CC, and we see a similar pattern in a PC. We emphasize that we are not comparing the performances between a PC and CC, however, we use the PC to verify the results and trends that we observe when using the CC.

## 4. Conclusions

We are interested in developing an object detection module that will detect good and bad landing spots in real-time, while working on a companion computer that is attached to a UAV. From previous related work, we could not conclude the object detection algorithm that works best in this application, while satisfying the safety requirement that we desire. Therefore, we selected YOLOv3, YOLOv4, and YOLOv5l due to their good detection speed and accuracy in real-time applications, and compare their accuracy and speed to investigate which algorithm performs best for emergency landing spot detection. We utilized the DOTA aerial image data set for training, testing and validation, and then tested the YOLO algorithms on a CC. From the results of our investigations, presented in Table 3 and Figure 5 we can confirm that all the three algorithms satisfy the safety requirement on the CC. Therefore, based on Table 3 and Figure 5 we choose the algorithm with the highest accuracy, and that is YOLOv5l. Hence, we can confirm the feasibility of using YOLOv5l with the DOTA dataset for emergency landing spot detection with sufficient speed and accuracy. We also tested the algorithms on a PC and verified the results that we got on the CC with the results from the PC. We conclude that the YOLOv5l algorithm with DOTA dataset is the best option among the three algorithms that can help with detecting emergency landing spots in real-time, while ensuring the safety requirements. 

## Figures and Tables

**Table 1:** Comparison of YOLO with related works.

Reference	Dataset Used	Algorithms	Findings
Li et al., 2021 [26]	Remote sensing images collected from GF-1 and GF-2 satellites. Training: 826 images. Testing: 275 images. Resolution: 300 × 300, 416 × 416, 500 × 500, 800 × 800, 1000 × 1000	Faster R-CNN YOLO v3 SSD	YOLOv3 has higher mAP and FPS than SSD and Faster R-CNN algorithms.
Benjdira et al., 2019 [12]	UAV dataset Training: 218 Images Test: 52 Images Resolution: 600 × 600 to 1024 × 1024	Faster R-CNN YOLOv3	YOLOv3 has higher F1 score and FPS than Faster R-CNN.
Zhao et al., 2019 [27]	Google Earth and DOTA datasetTraining: 224 Images Test: 56 Images Resolution: 600 × 600 to 1500 × 1500	SSD Faster R-CNN YOLOv3	YOLOv3 has higher mAP and FPS than Faster R-CNN and SSD.
Kim et al., 2020 [29]	Korea expressway dataset Training: 2620 Test: 568 Resolution: NA	YOLOv4 SSD Faster R-CNN	YOLOv4 has higher accuracy SSD has higher detection speed
Dorrer et al., [28]	Custom Refrigerator images Training: 800 Images Test: 70 Images Resolution: NA	Mask RCNN YOLOv3	The detection of YOLOv3 was 3 times higher but the accuracy of Mask RCNN was higher.
Rahman et al., [13]	Custom Electrical dataset Training: 5939 Test: 1400 Resolution: NA	YOLOv4 YOLOv5l	YOLOv4 has higher mAP compared to YOLOv5l algorithms
Long et al., [30]	MS COCO dataset Training: 118,000 Test: 5000 Resolution: NA	YOLOv3 YOLOv4	YOLOv4 has higher mAP compared to YOLOv3
Bochkovskiy et al., [7]	MS COCO dataset Training: 118,000 Test: 5000 Resolution: NA	YOLOv3 YOLOv4	YOLOv4 has higher mAP and fps than YOLOv3
Ge et al., [14]	MS COCO dataset Training: 118,000 Test: 5000 Resolution: NA	YOLOv3 YOLOv4 YOLOv5	YOLOv5 has higher mAP than YOLOv3 and YOLOv5l YOLOv3 has higher FPS than YOLOv4 and YOLOv5l

**Table 2:** Comparison between structures of YOLOv3, YOLOv4 and YOLOv5.

	YOLOv3	YOLOv4	YOLOv5
Neural Network Type	Fully convolution	Fully convolution	Fully convolution
Backbone Feature Extractor	Darknet-53	CSPDarknet53	CSPDarknet53
Loss Function	Binary cross entropy	Binary cross entropy	Binary cross entropy and Logits loss function
Neck	FPN	SSP and PANet	PANet
Head	YOLO layer	YOLO layer	YOLO layer

**Table 3:** Results of comparing YOLOv3, YOLOv4 and YOLOv5l algorithms for emergency landing spot detection.

Measure	YOLOv3	YOLOv4	YOLOv5l
Precision	0.73	0.69	0.707
Recall	0.41	0.57	0.611
F1 Score	0.53	0.63	0.655
mAP	0.46	0.607	0.633
PC Speed (FPS)	63.7	59	58.82
Jetson Speed (FPS)	7.5	6.8	5

## Supplementary Materials

The following supporting information can be downloaded at: https://www.mdpi.com/article/10.3390/s22020464/s1, This YouTube video: youtu.be/q2ljifrQkwE shows the output of the chosen algorithm (YOLOv5l) working on a video stream of a UAV flying near Southern Illinois University campus. We can see in the video that the algorithm can detect places to land and places to avoid. In addition, the Images folder inside this link: tinyurl.com/Sensors1498487 includes three folders; YOLOv3, YOLOv4, and YOLOv5l, each of which contains several outputs of YOLOv3, YOLOv4 and YOLOv5l algorithms, respectively.

## Appendix A

**Table A1:** Performance of YOLOv3, YOLOv4, and YOLOv5l.

Label	YOLOv3 Average Precision	YOLOv4 Average Precision	YOLOv5l Average Precision
Small-Vehicle	29.25	39.62	44.8
Large-Vehicle	55.84	73.43	70.1
Plane	83.06	90.39	91.3
Storage-tank	44.69	61.52	63
Ship	71.19	82.67	78.6
Harbor	67.94	80.35	82.7
Ground-track-field	36.12	67.32	65.7
Soccer-ballfield	36.82	54.24	59.8
Tennis-court	87.30	92.57	92.7
Swimming-pool	39.76	57.57	65.4
baseball	61.35	76.62	75.8
roundabout	44.14	55.98	55.9
Basketball-court	37.79	63.04	64.5
bridge	26.65	42.41	50.1
helicopter	15.84	34.54	48.2
