# Supplementary figures and images for: Comparing YOLOv3, YOLOv4 and YOLOv5 for Autonomous Landing Spot Detection in Faulty UAVs
Source: Sensors (Basel). 2022 Jan 8;22(2):464. doi: 10.3390/s22020464 (PMC8778480; doi:10.3390/s22020464)

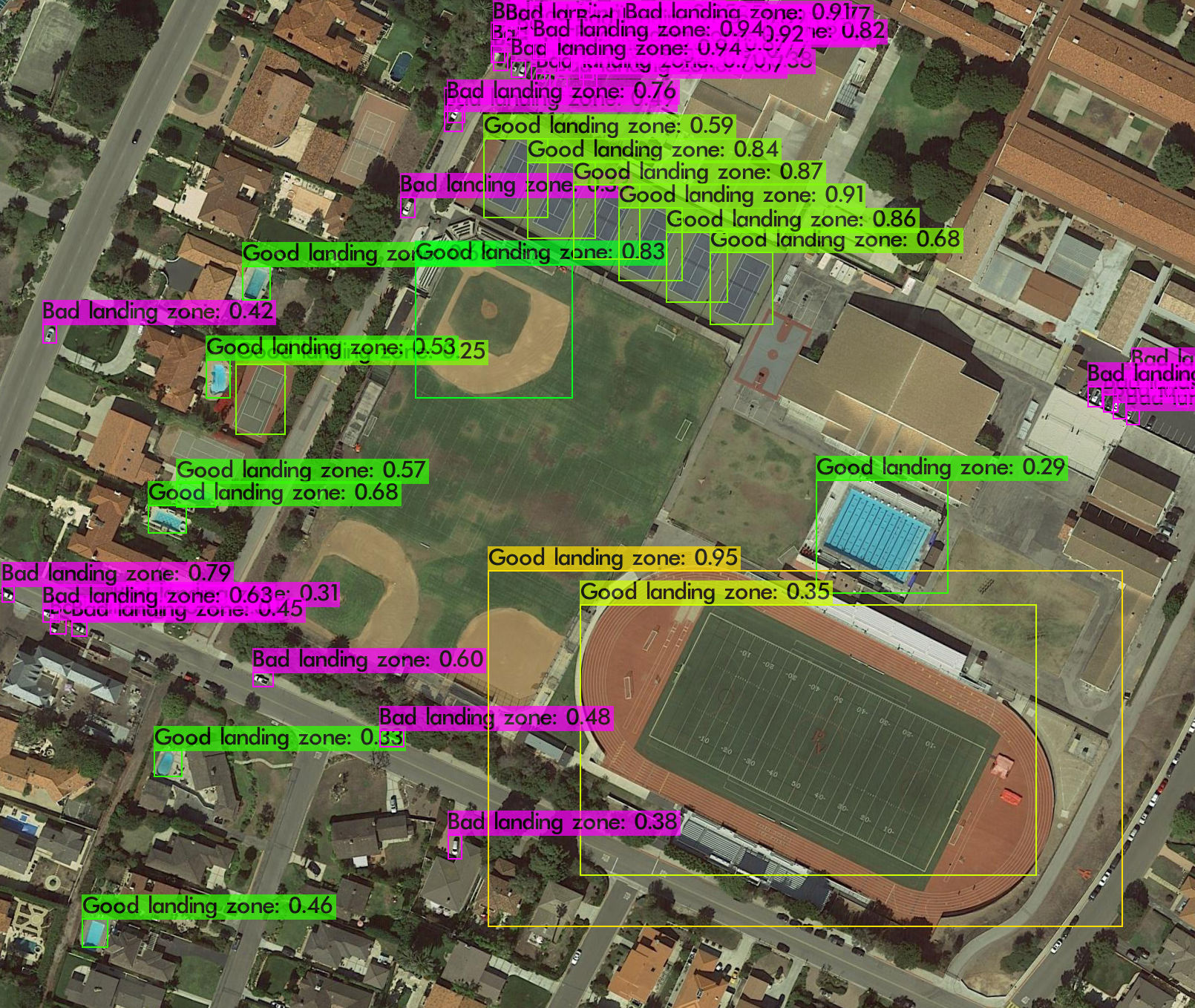

Supplement: Supplementary file 1 [file sensors-22-00464-s001.zip › Images/YOLOv3/Picture1.jpg]

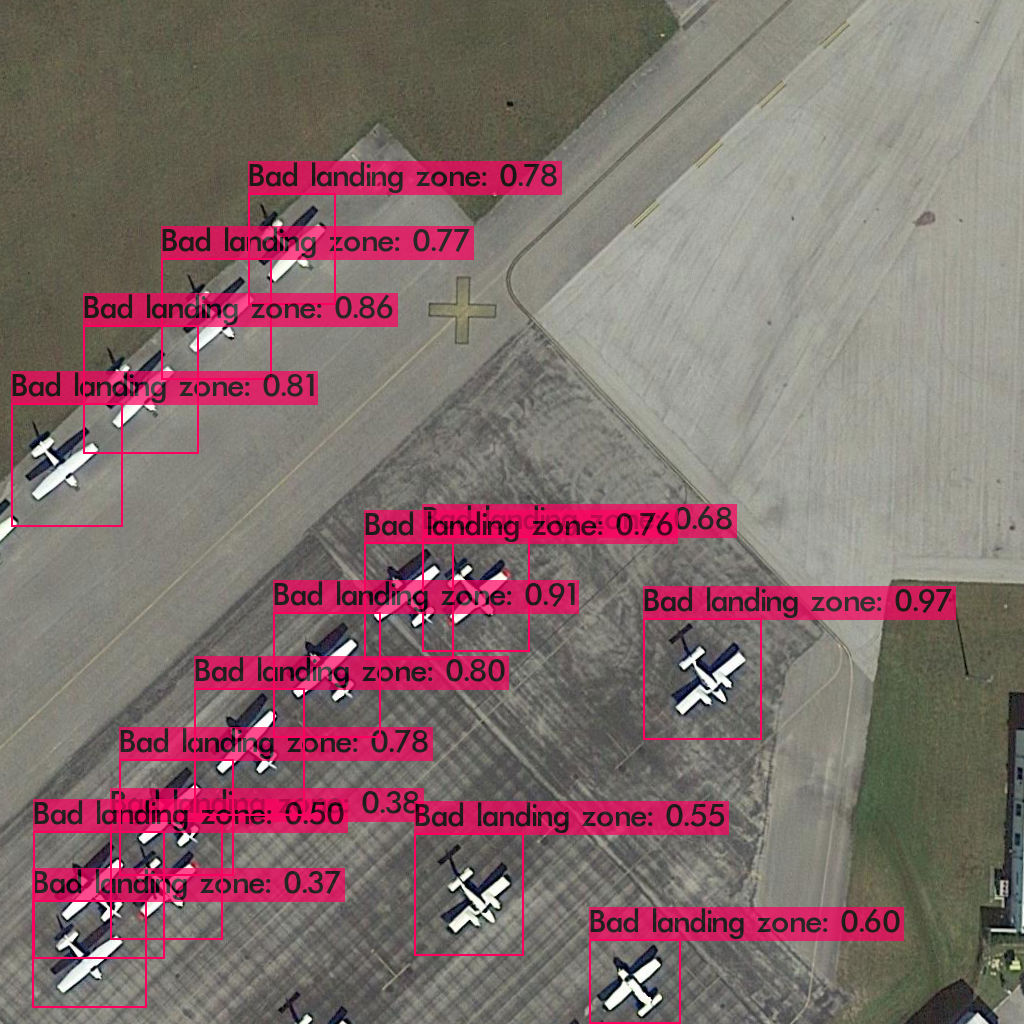

Supplement: Supplementary file 1 [file sensors-22-00464-s001.zip › Images/YOLOv3/Picture10.jpg]

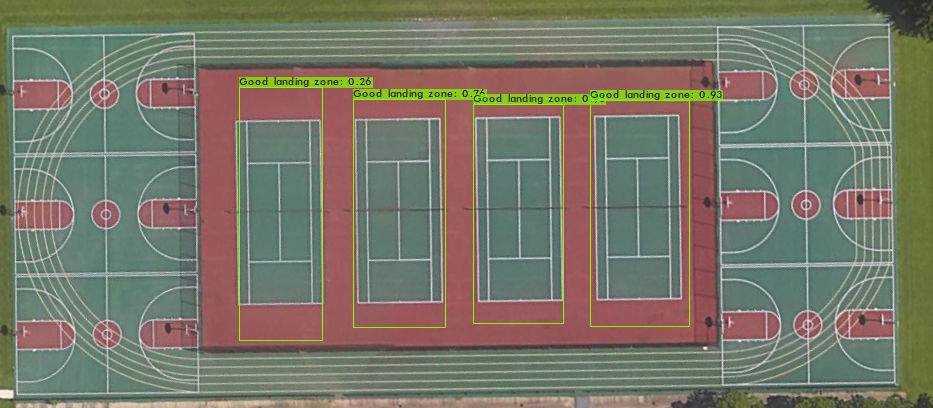

Supplement: Supplementary file 1 [file sensors-22-00464-s001.zip › Images/YOLOv3/Picture2.jpg]

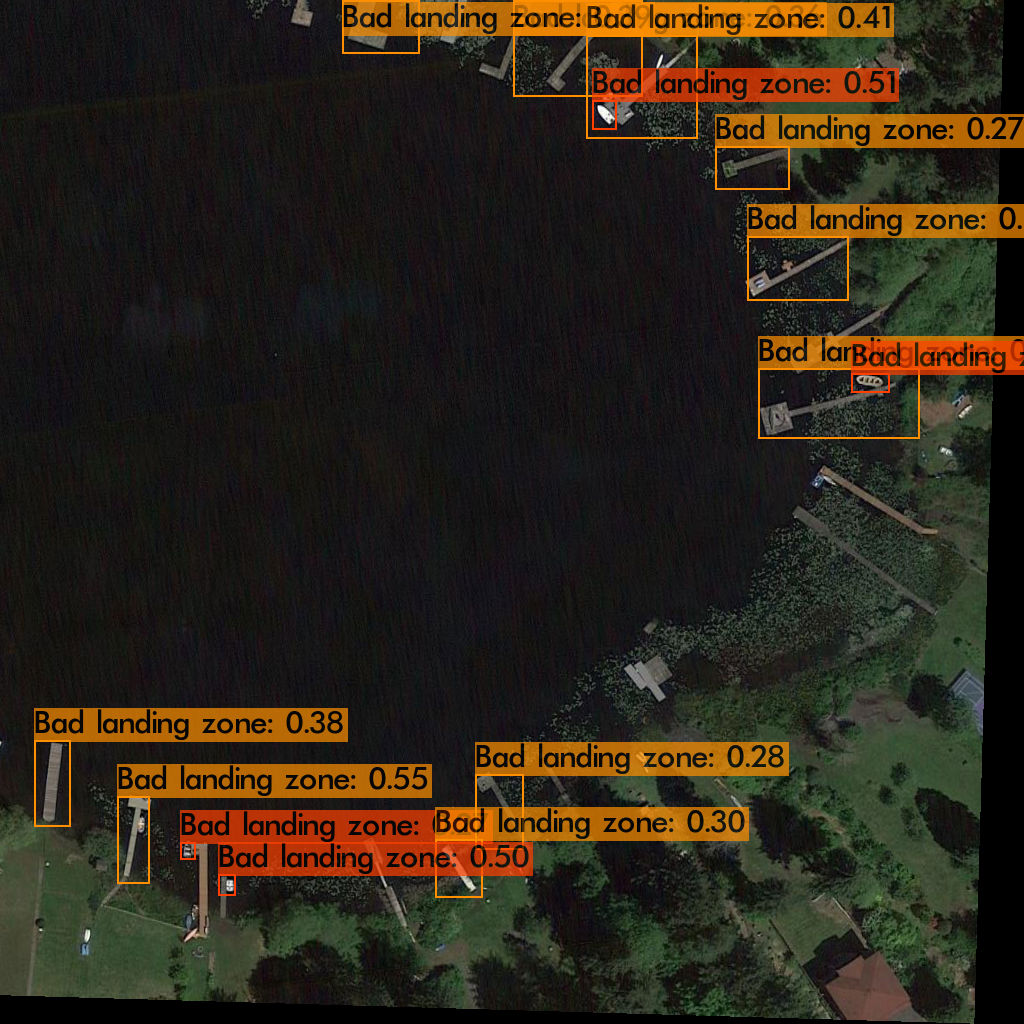

Supplement: Supplementary file 1 [file sensors-22-00464-s001.zip › Images/YOLOv3/Picture3.jpg]

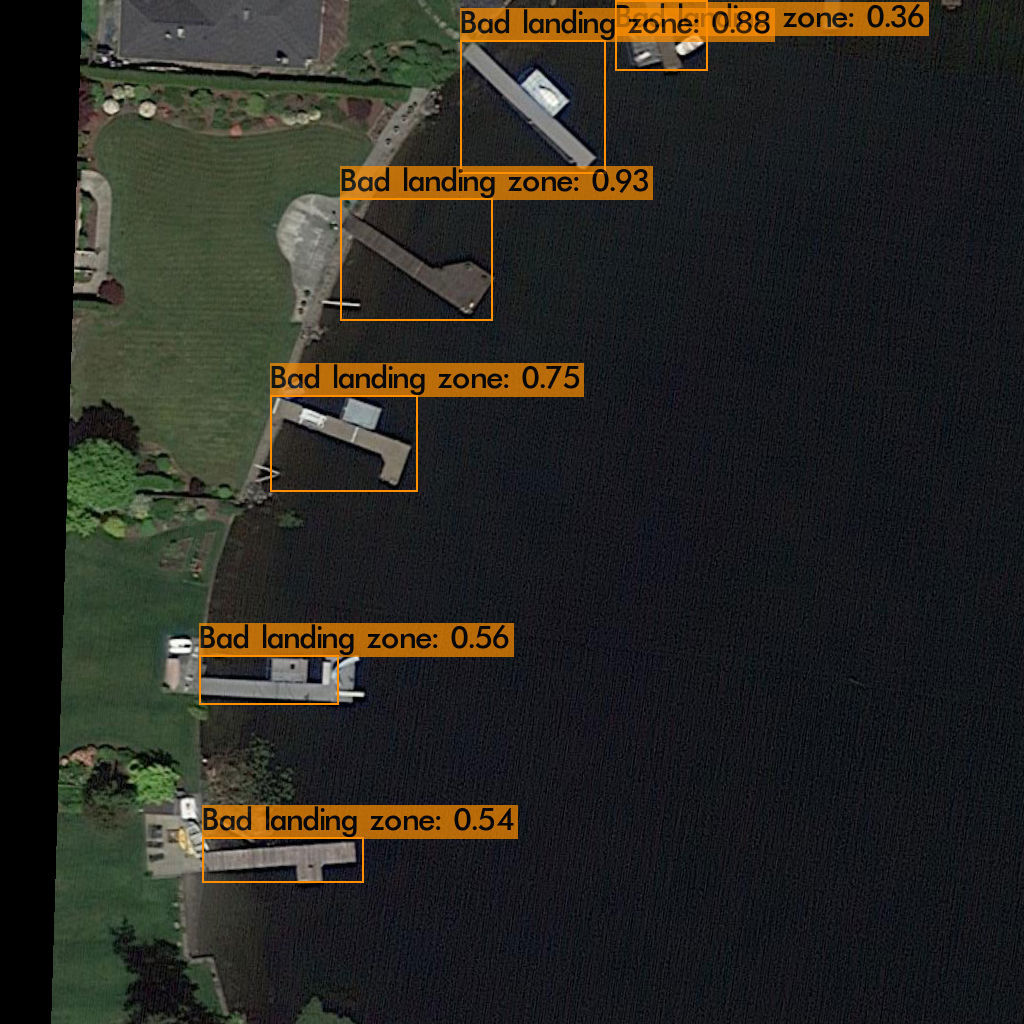

Supplement: Supplementary file 1 [file sensors-22-00464-s001.zip › Images/YOLOv3/Picture4.jpg]

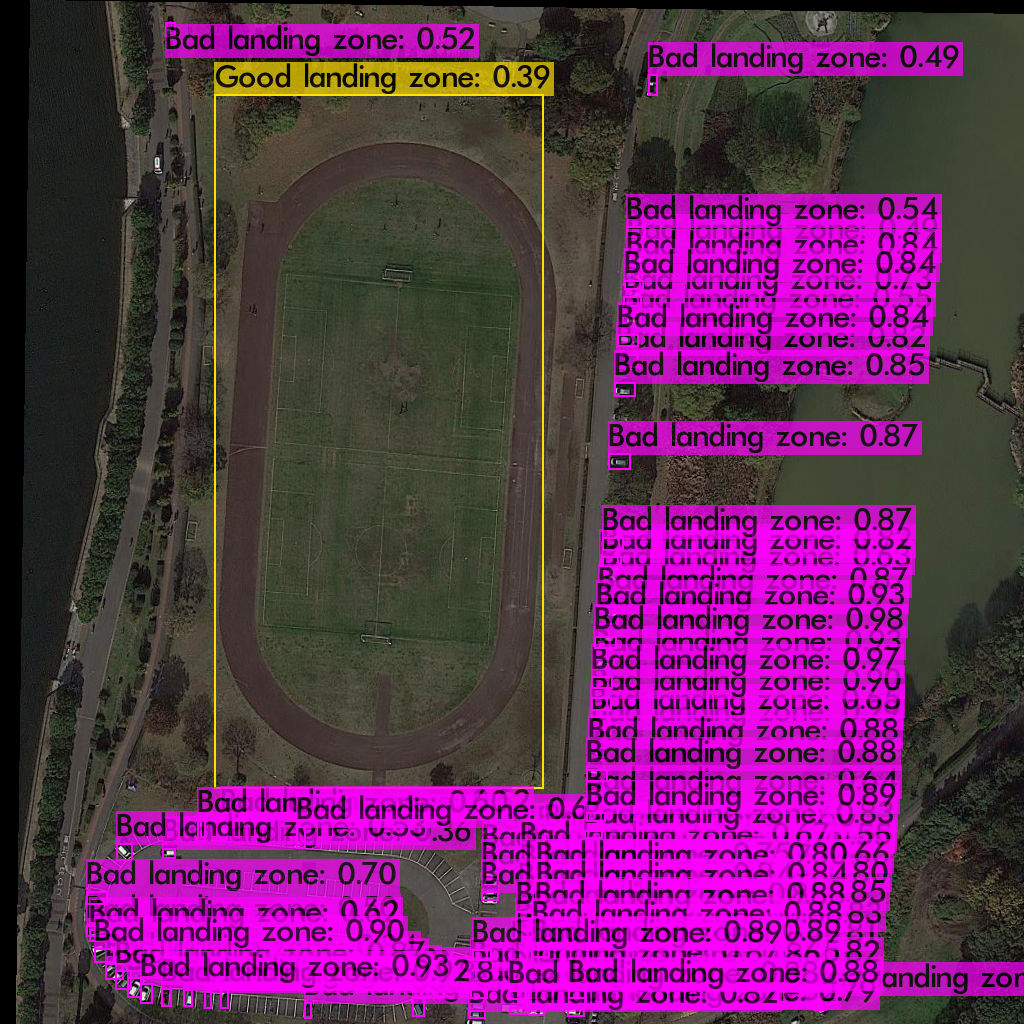

Supplement: Supplementary file 1 [file sensors-22-00464-s001.zip › Images/YOLOv3/Picture5.jpg]

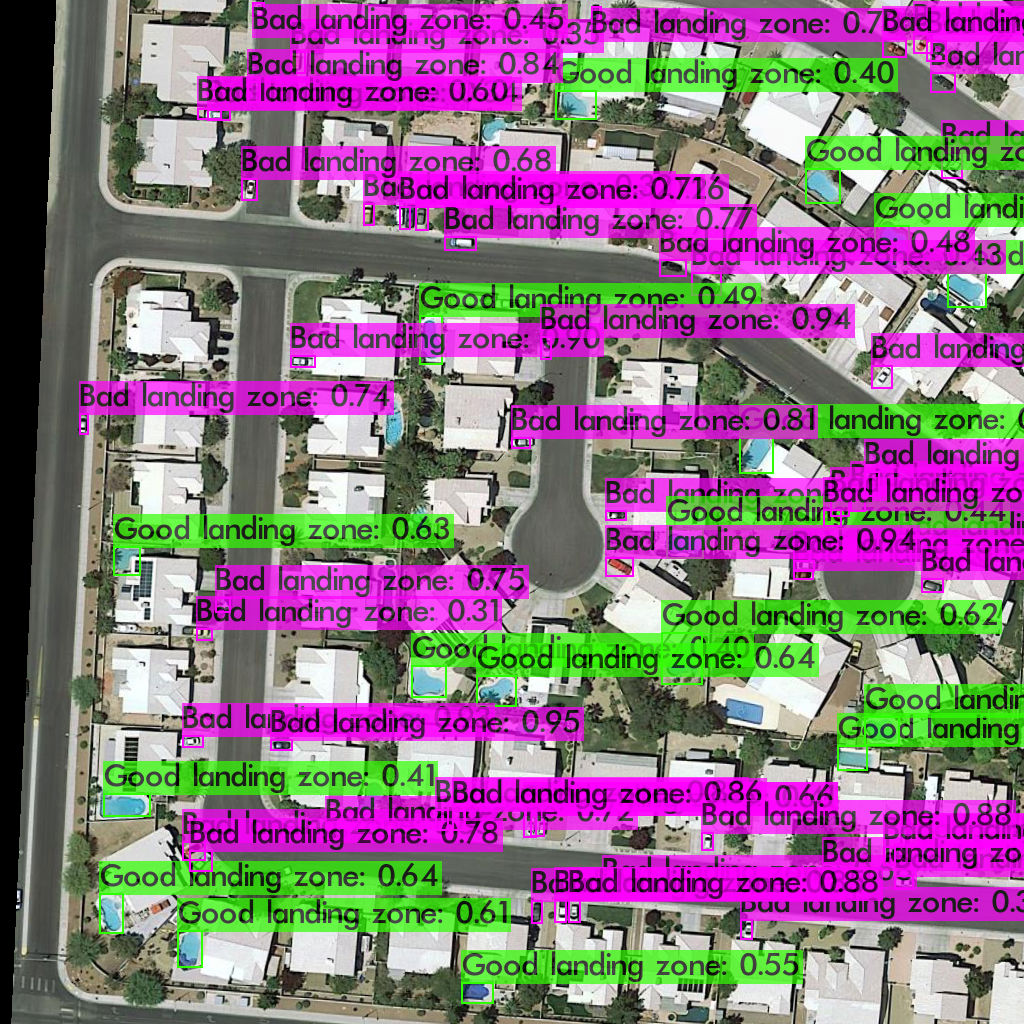

Supplement: Supplementary file 1 [file sensors-22-00464-s001.zip › Images/YOLOv3/Picture6.jpg]

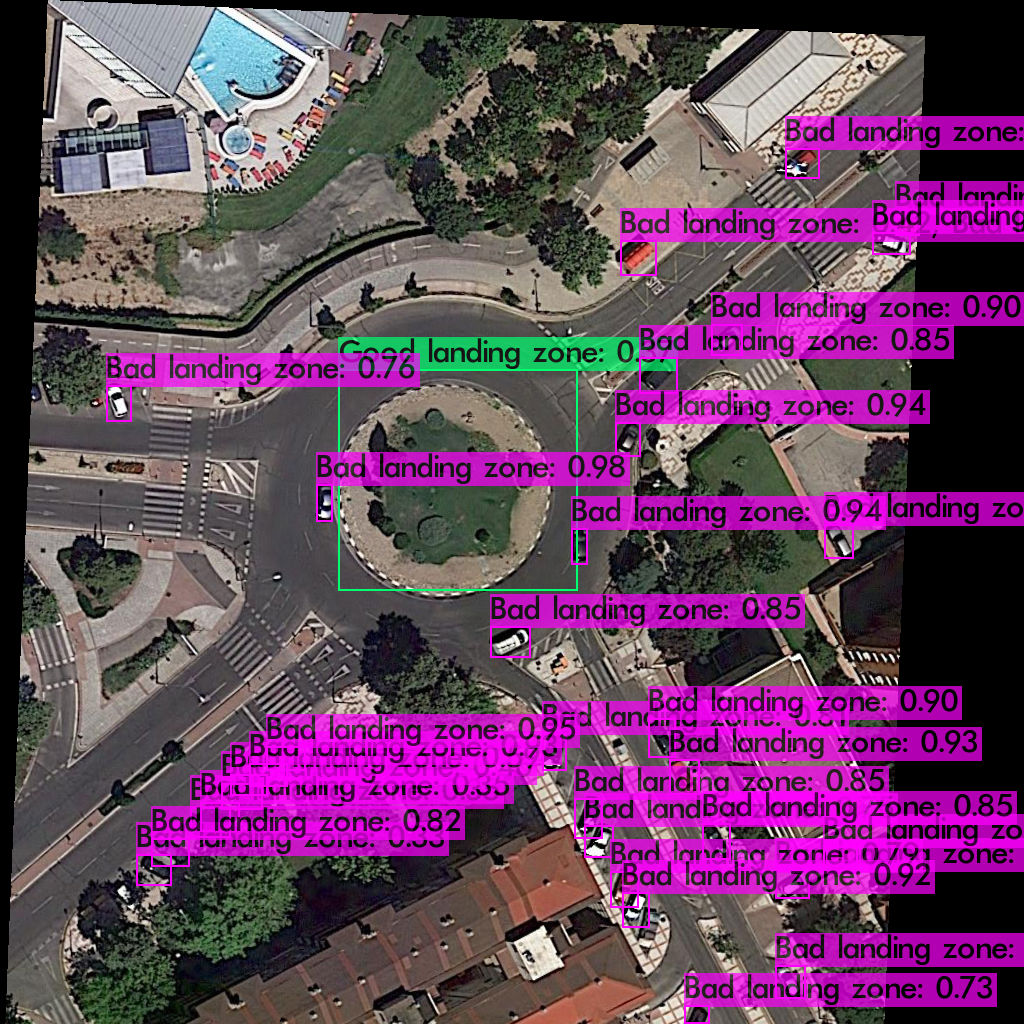

Supplement: Supplementary file 1 [file sensors-22-00464-s001.zip › Images/YOLOv3/Picture7.jpg]

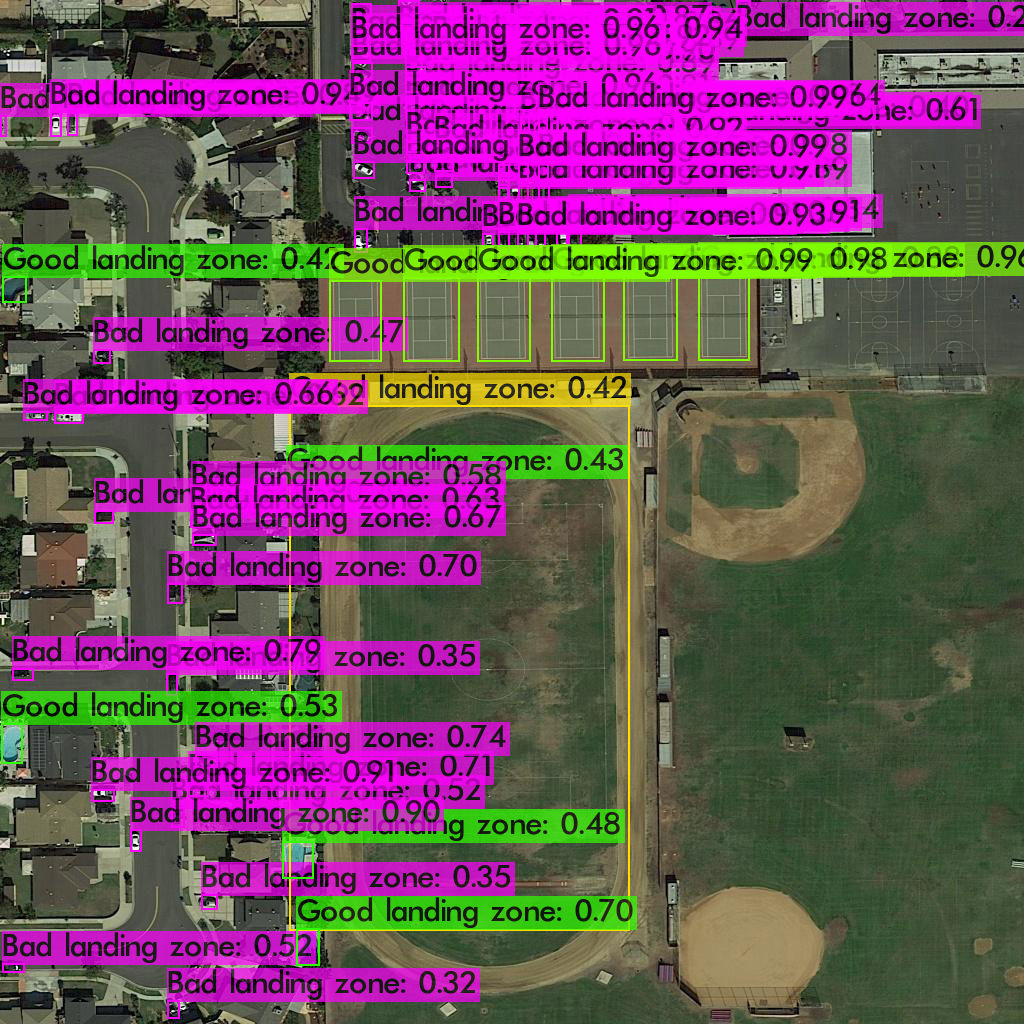

Supplement: Supplementary file 1 [file sensors-22-00464-s001.zip › Images/YOLOv3/Picture8.jpg]

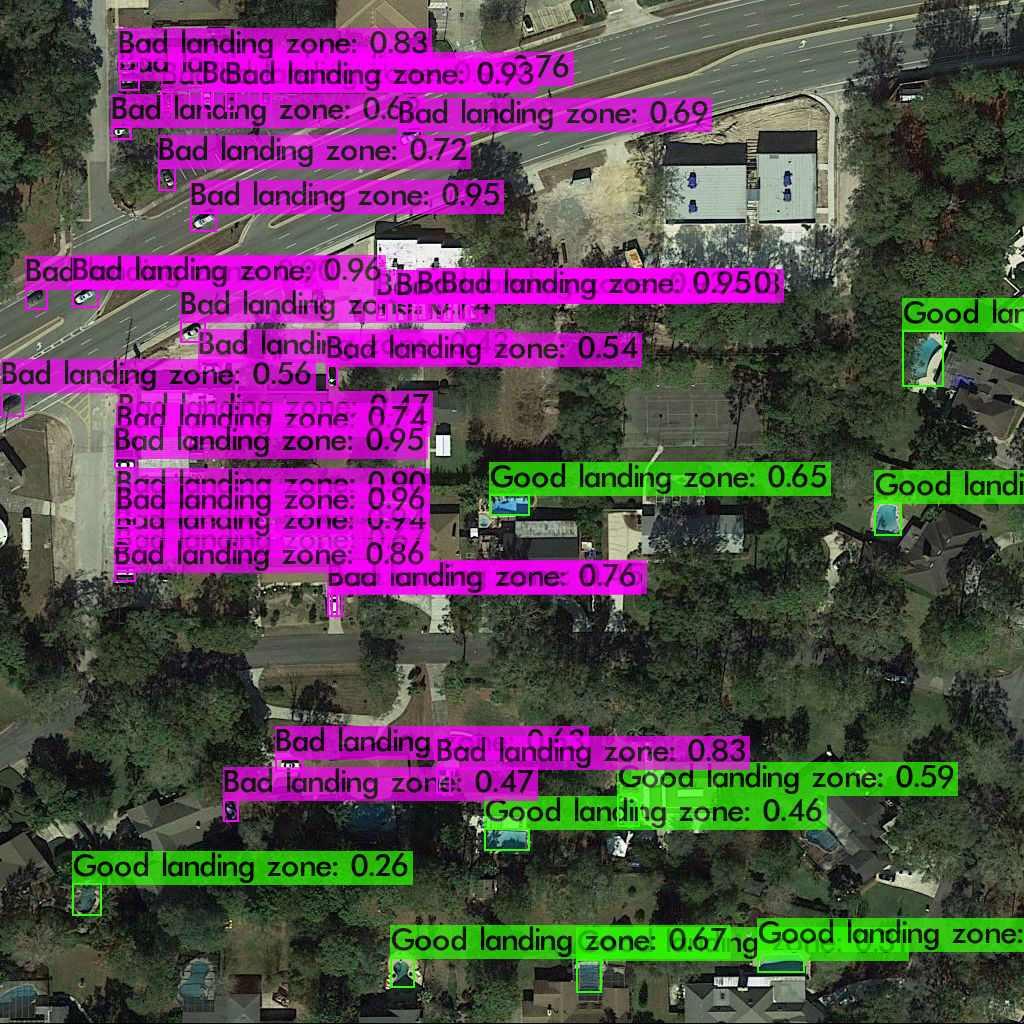

Supplement: Supplementary file 1 [file sensors-22-00464-s001.zip › Images/YOLOv3/Picture9.jpg]

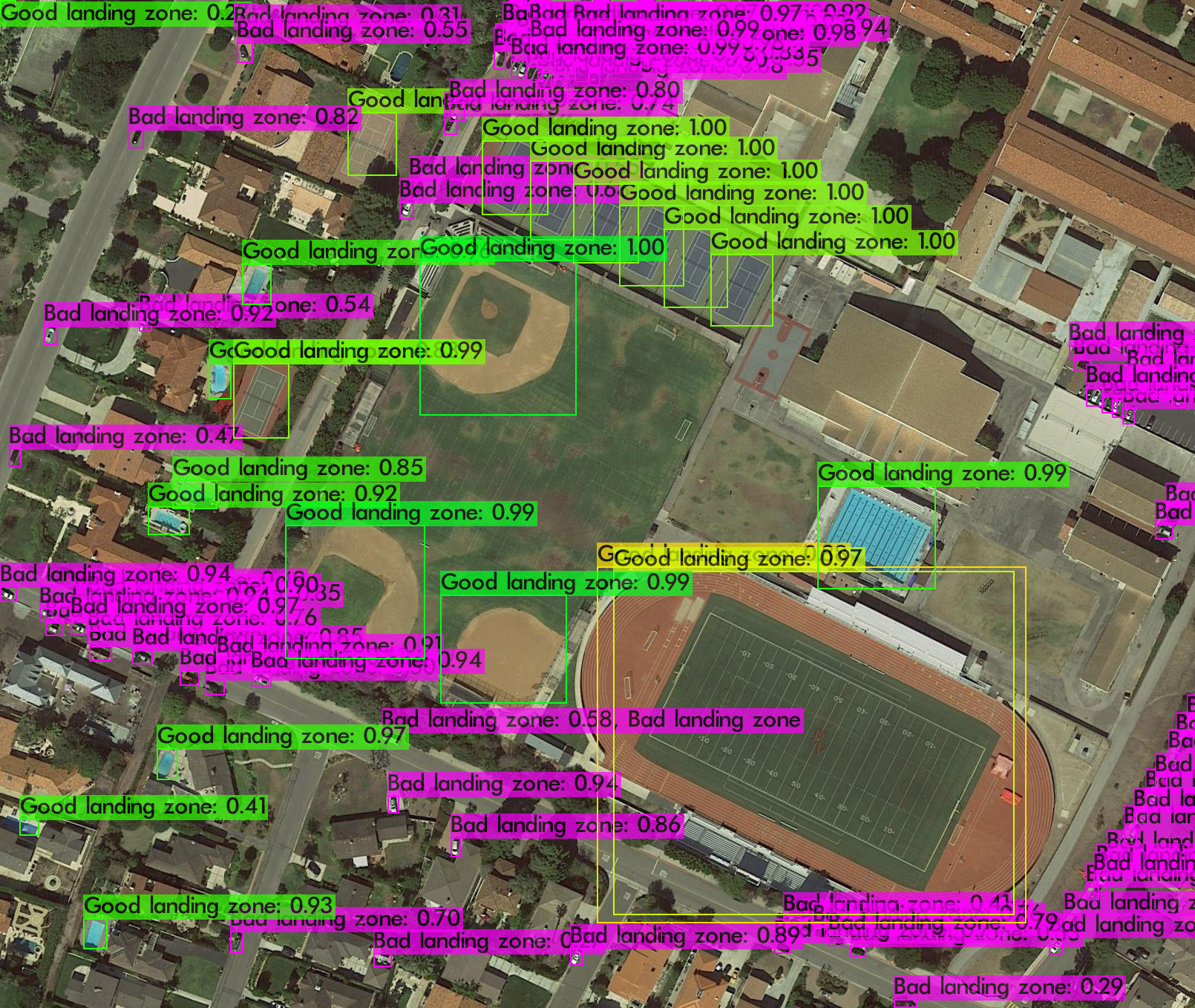

Supplement: Supplementary file 1 [file sensors-22-00464-s001.zip › Images/YOLOv4/Picture1.jpg]

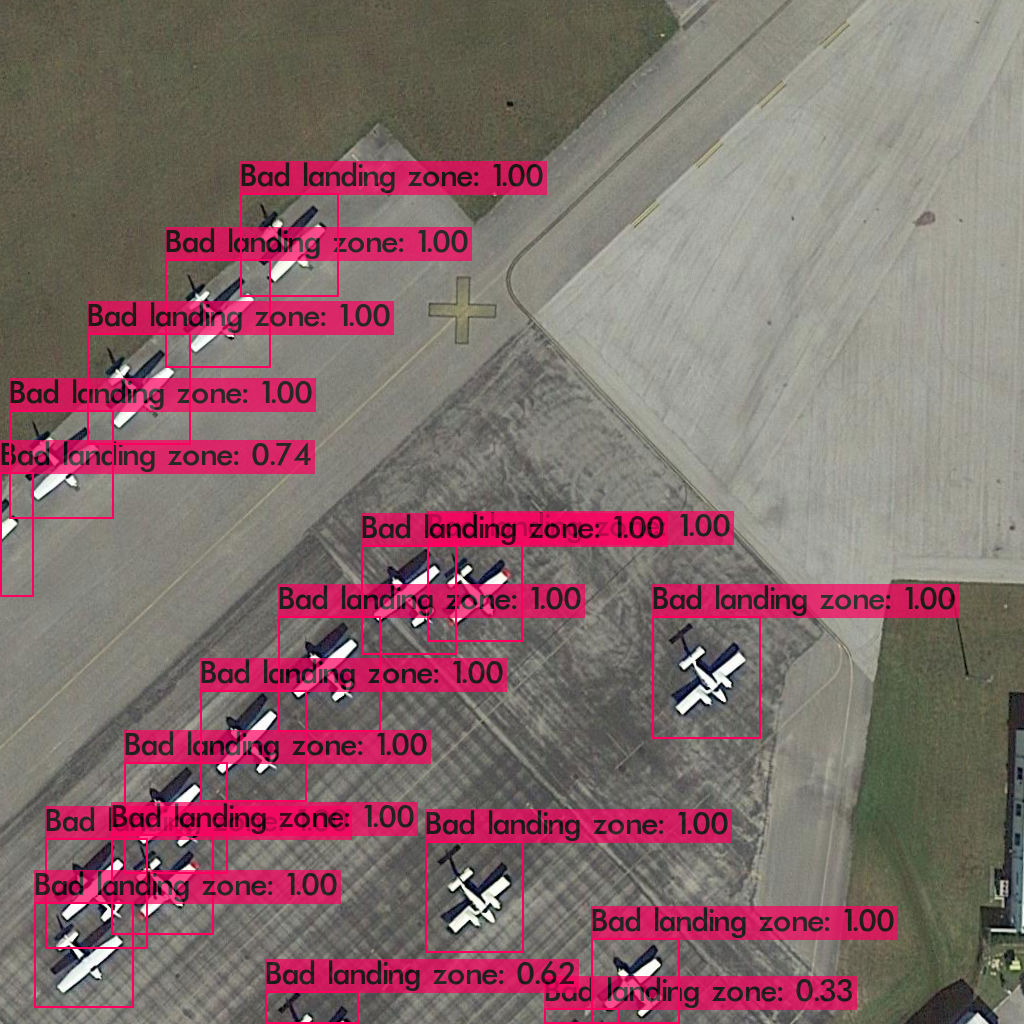

Supplement: Supplementary file 1 [file sensors-22-00464-s001.zip › Images/YOLOv4/Picture10.jpg]

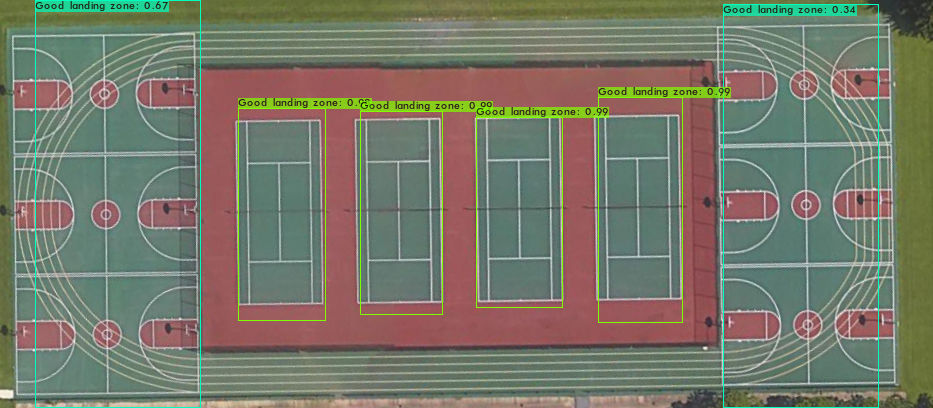

Supplement: Supplementary file 1 [file sensors-22-00464-s001.zip › Images/YOLOv4/Picture2.jpg]

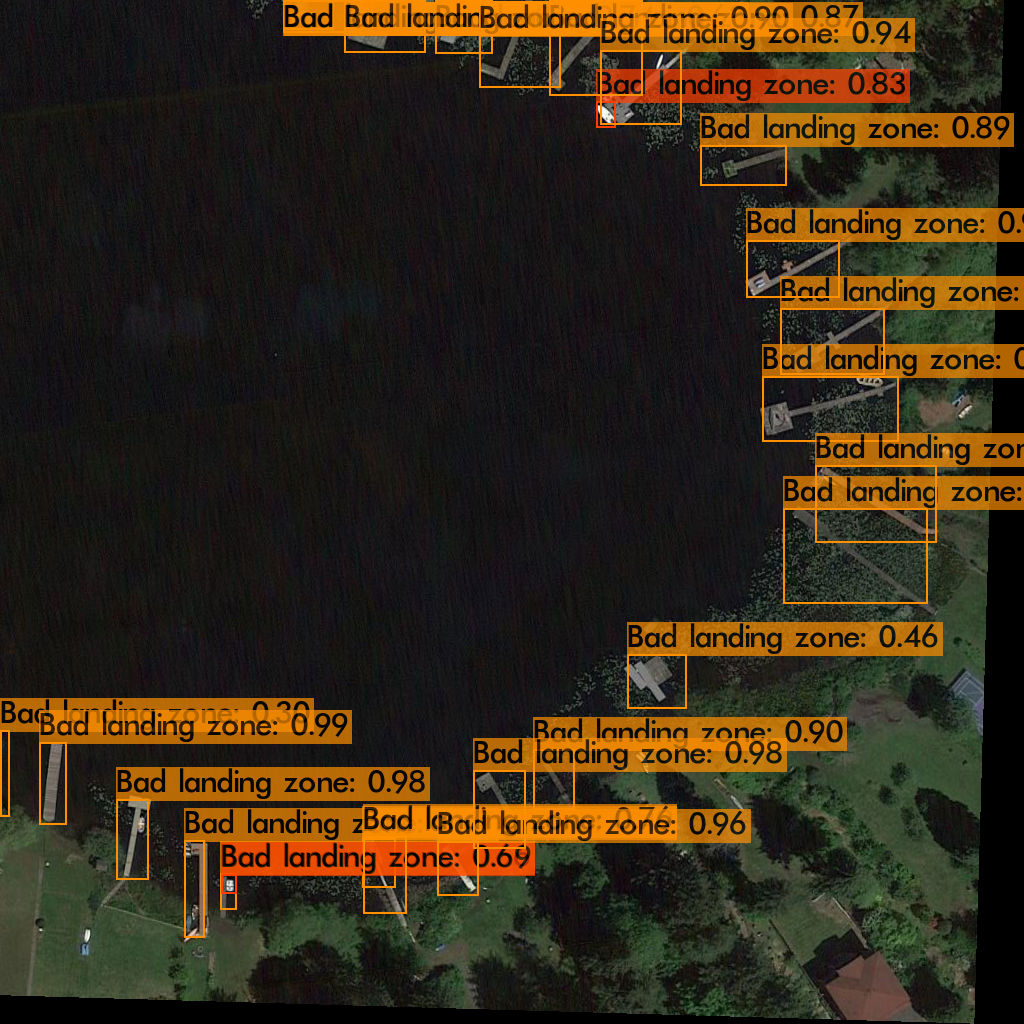

Supplement: Supplementary file 1 [file sensors-22-00464-s001.zip › Images/YOLOv4/Picture3.jpg]

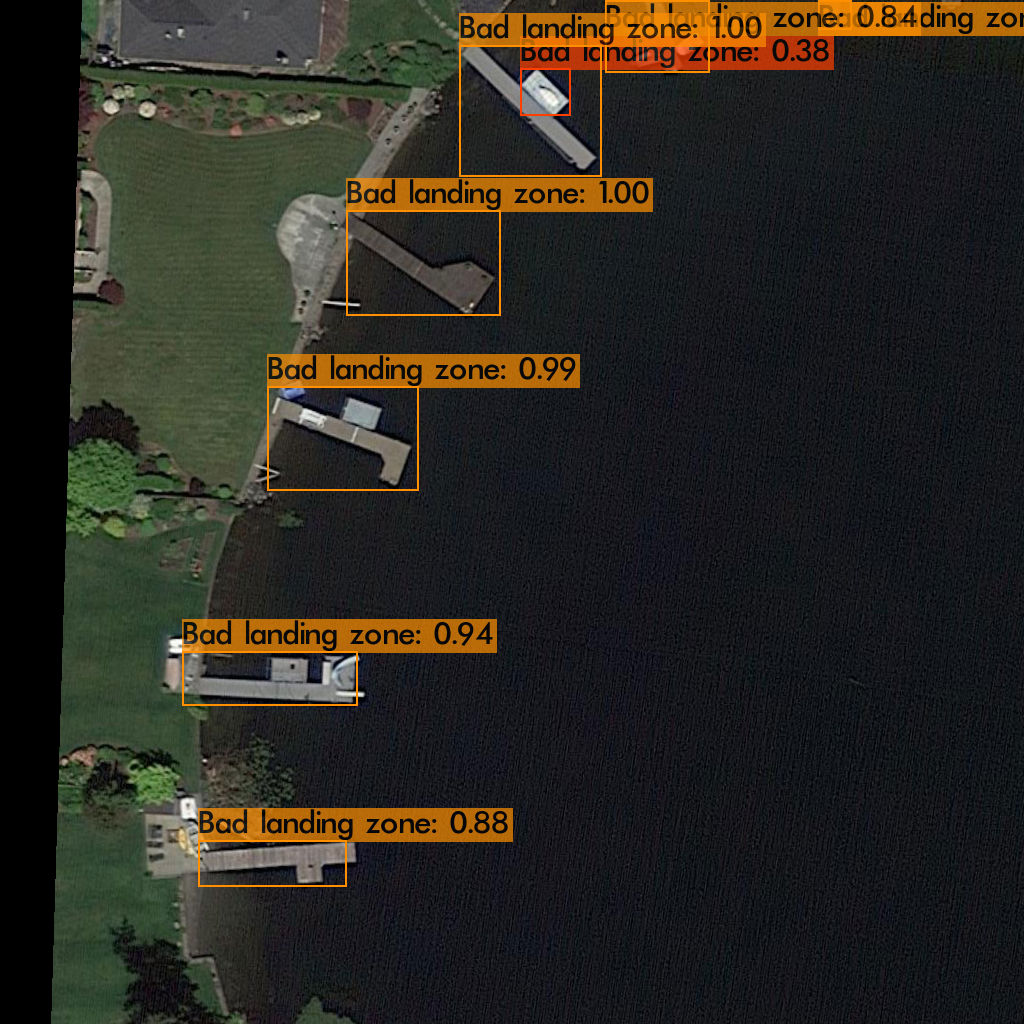

Supplement: Supplementary file 1 [file sensors-22-00464-s001.zip › Images/YOLOv4/Picture4.jpg]

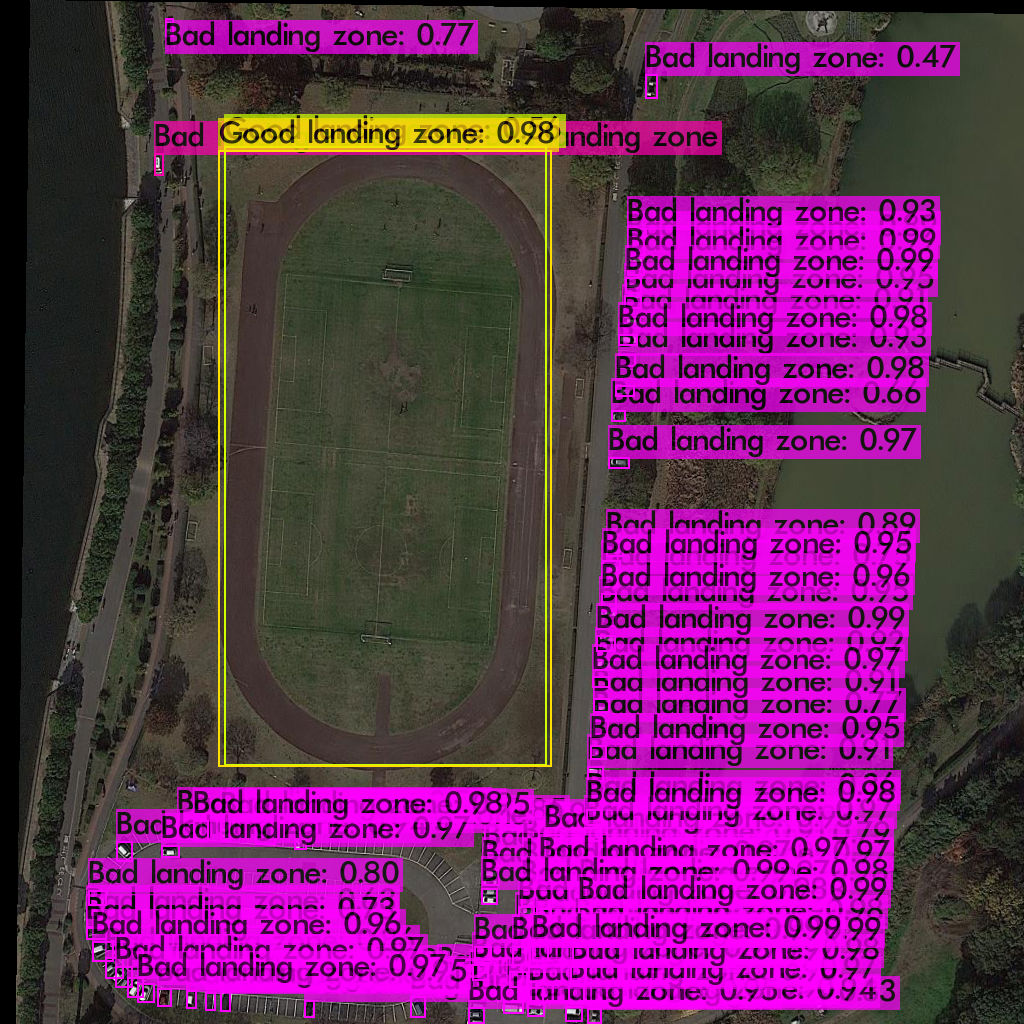

Supplement: Supplementary file 1 [file sensors-22-00464-s001.zip › Images/YOLOv4/Picture5.jpg]

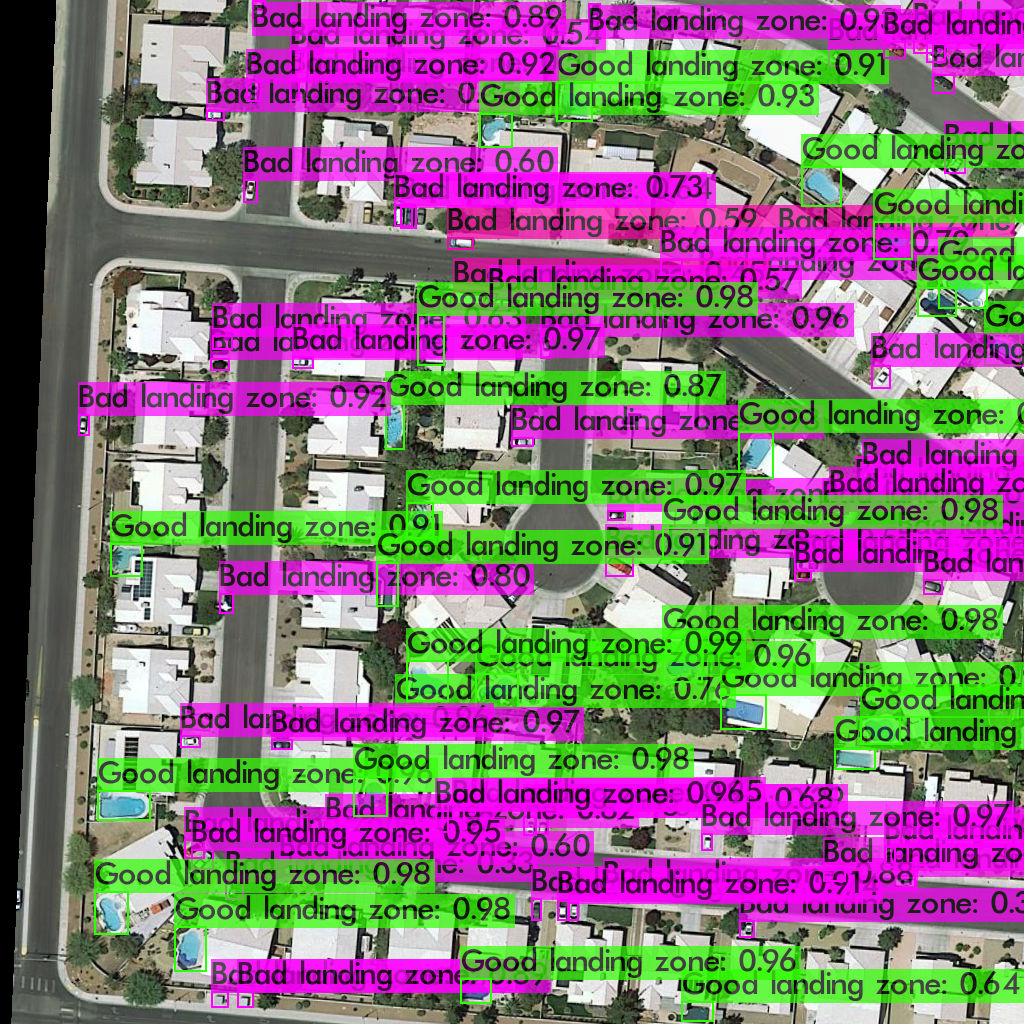

Supplement: Supplementary file 1 [file sensors-22-00464-s001.zip › Images/YOLOv4/Picture6.jpg]

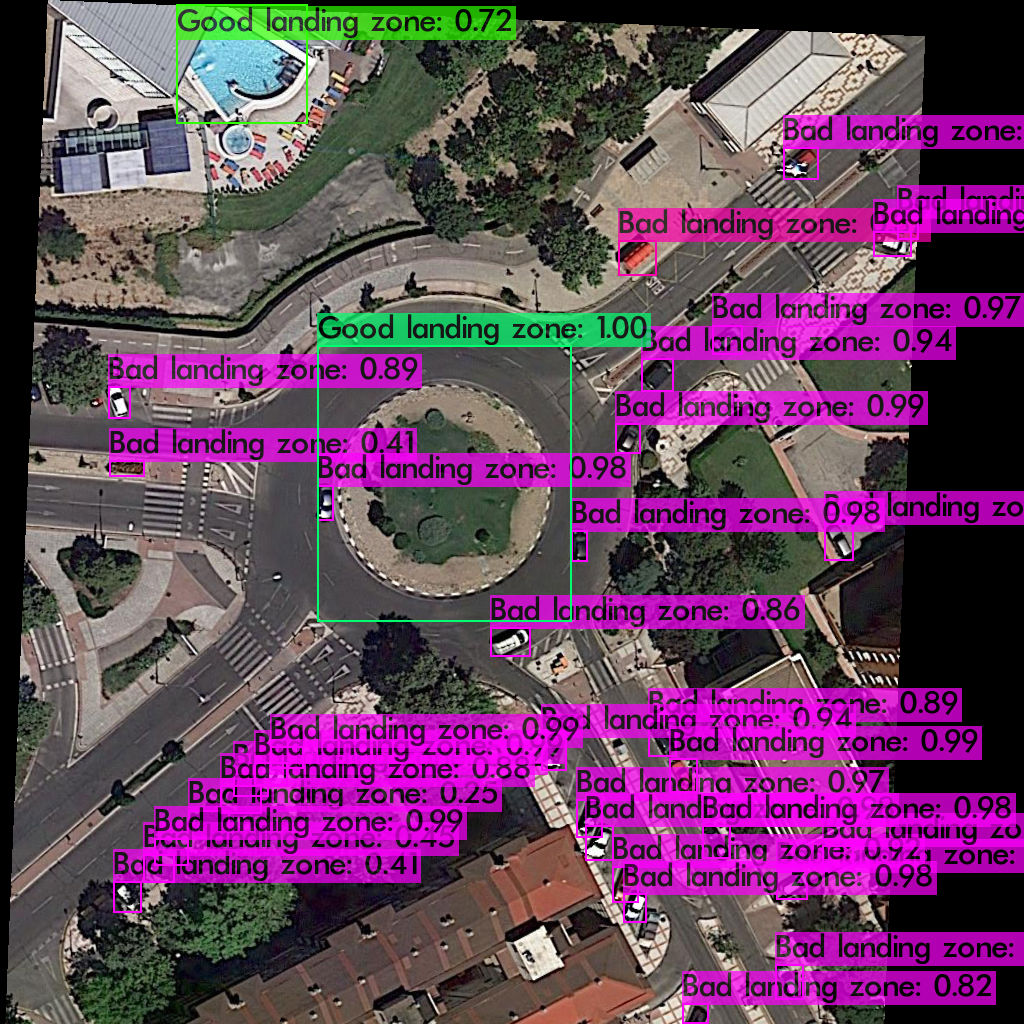

Supplement: Supplementary file 1 [file sensors-22-00464-s001.zip › Images/YOLOv4/Picture7.jpg]

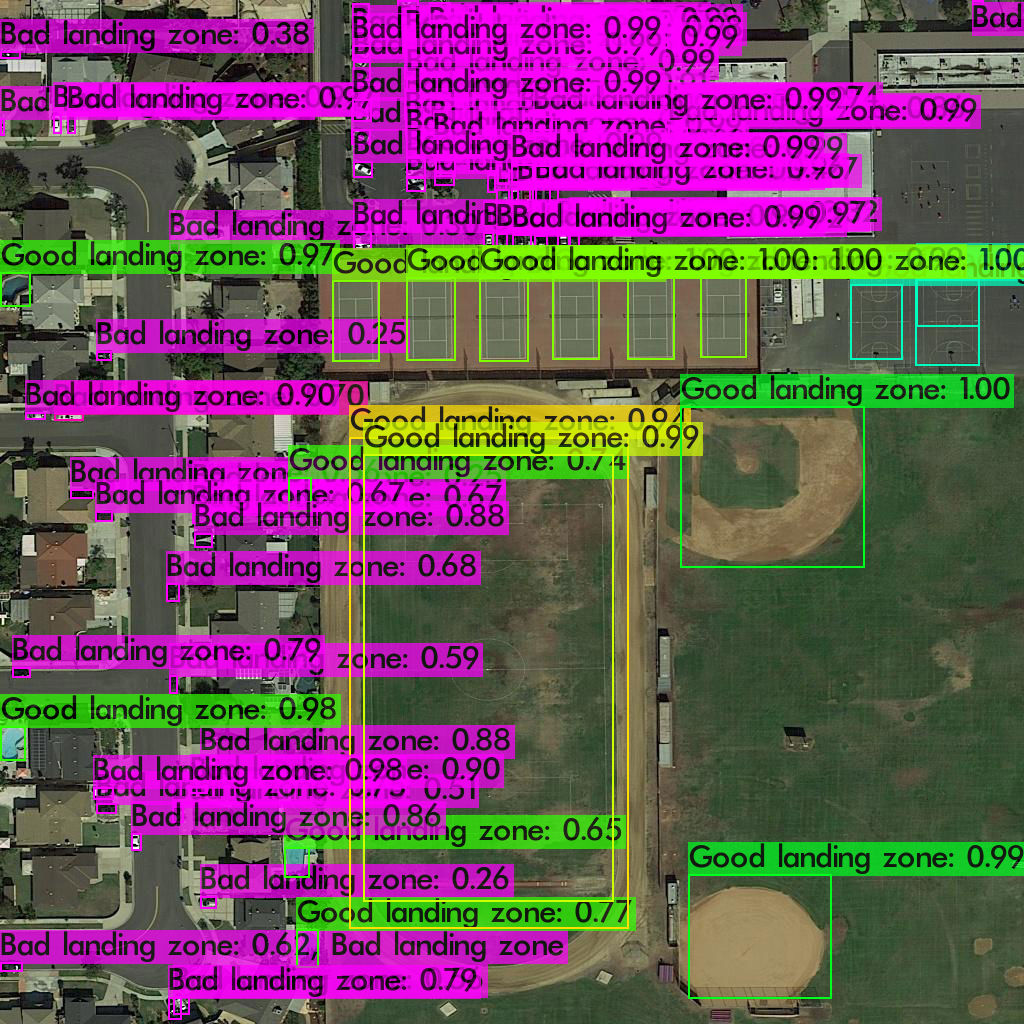

Supplement: Supplementary file 1 [file sensors-22-00464-s001.zip › Images/YOLOv4/Picture8.jpg]

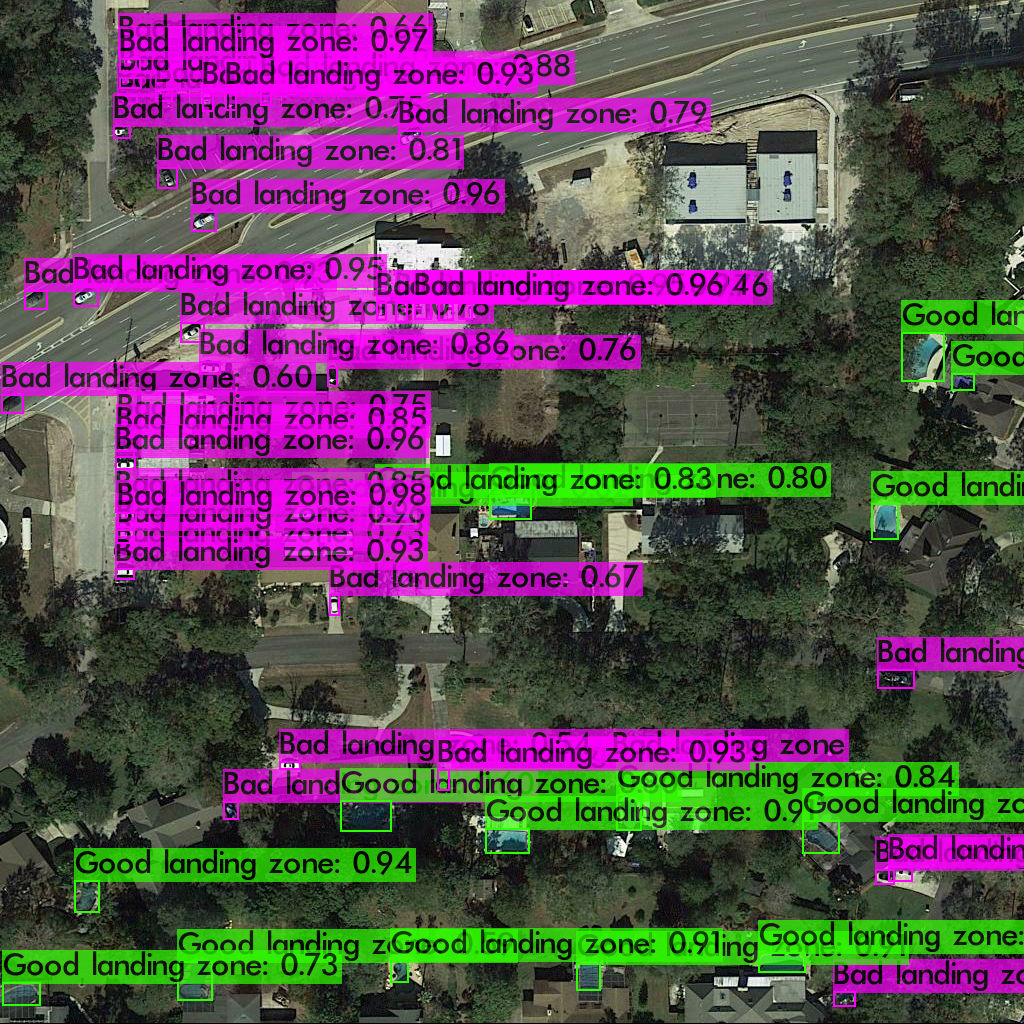

Supplement: Supplementary file 1 [file sensors-22-00464-s001.zip › Images/YOLOv4/Picture9.jpg]

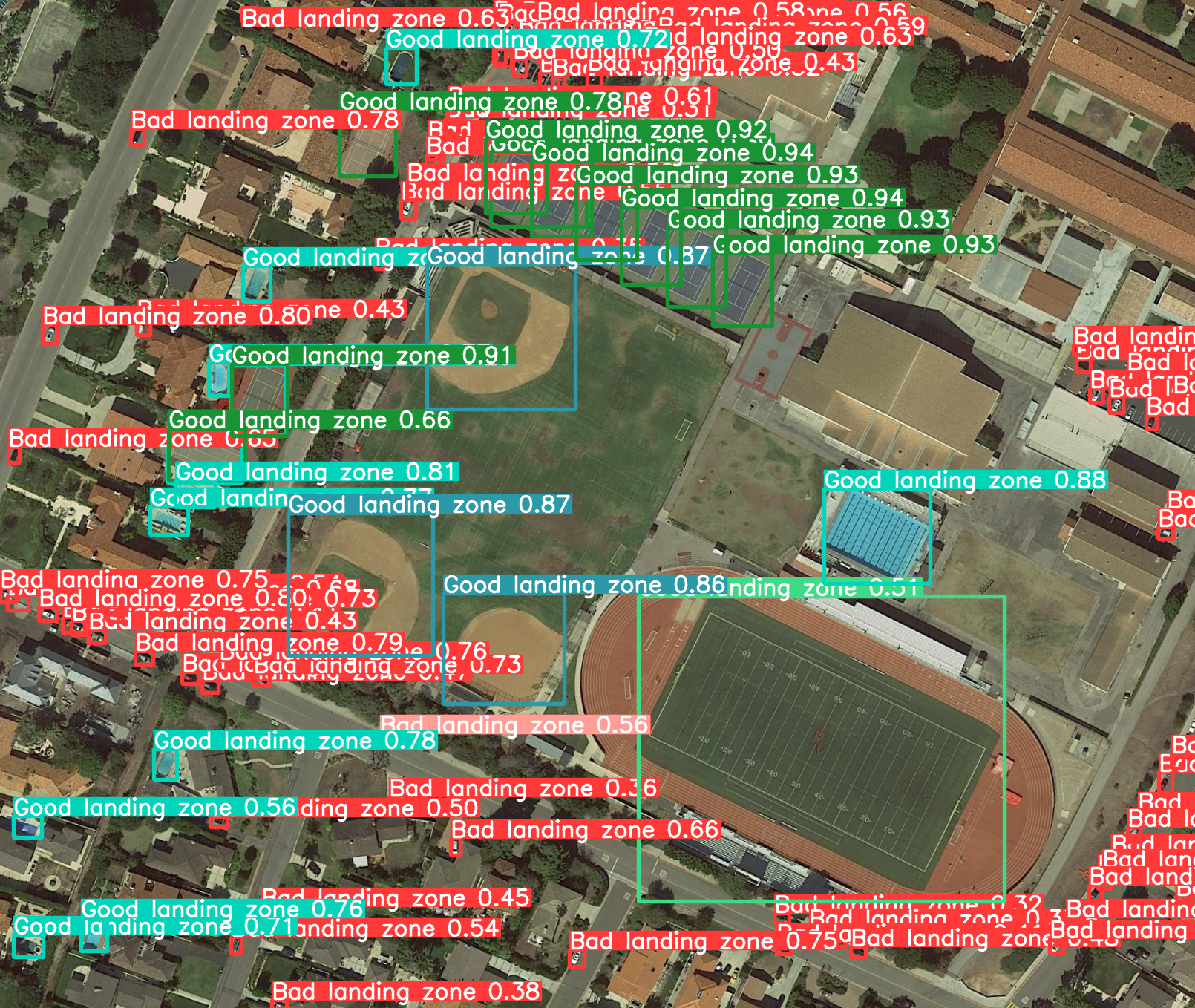

Supplement: Supplementary file 1 [file sensors-22-00464-s001.zip › Images/YOLOv5/Picture1.png]

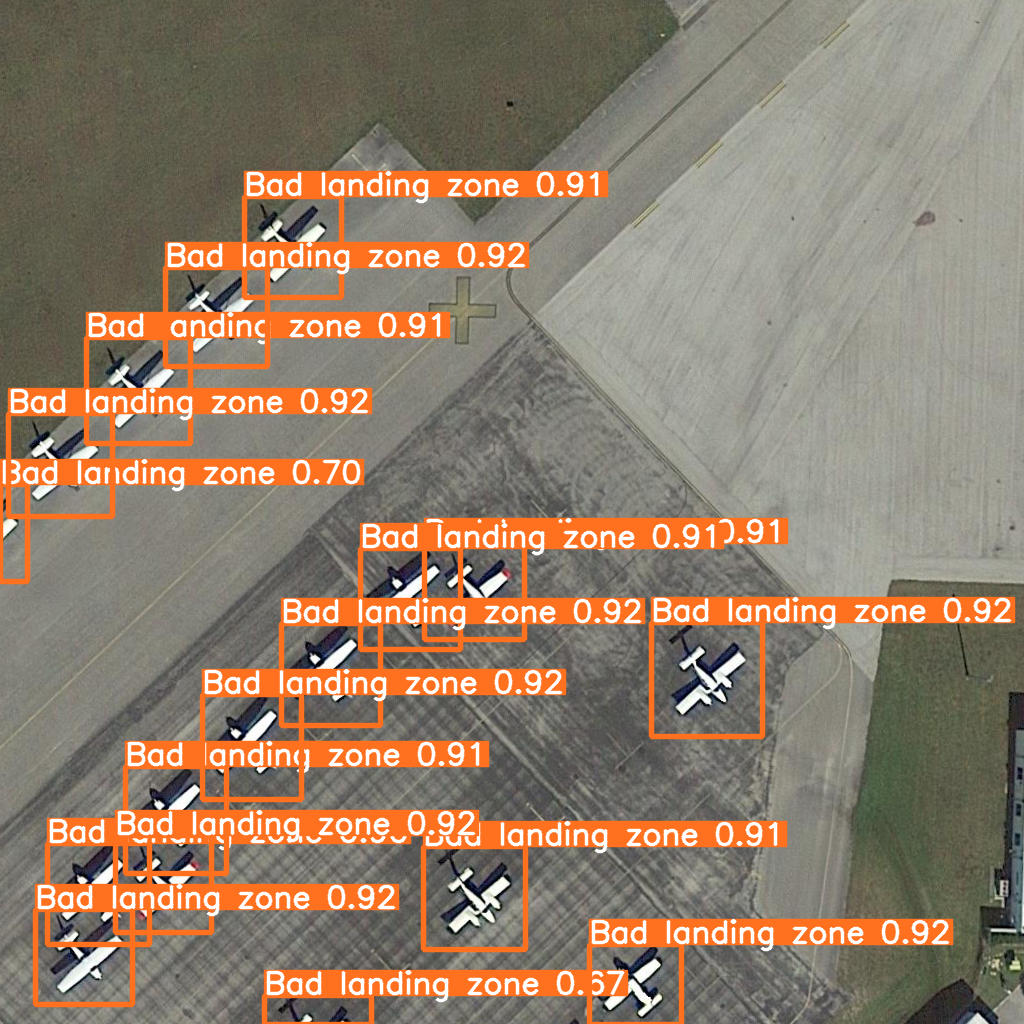

Supplement: Supplementary file 1 [file sensors-22-00464-s001.zip › Images/YOLOv5/Picture10.png]

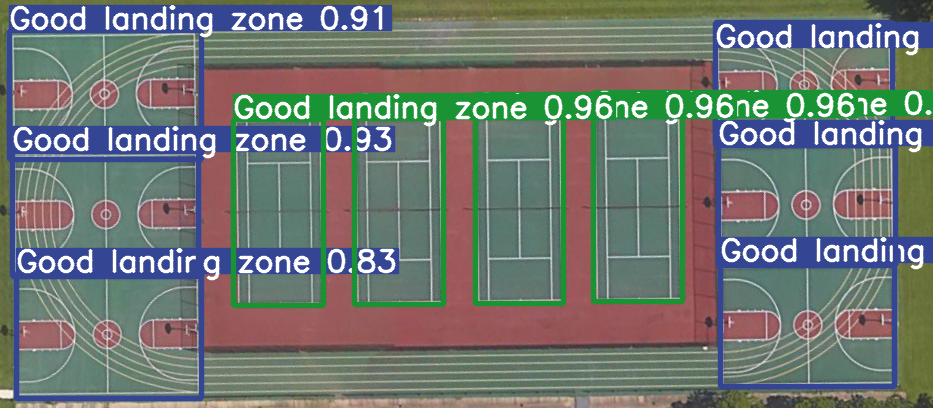

Supplement: Supplementary file 1 [file sensors-22-00464-s001.zip › Images/YOLOv5/Picture2.png]

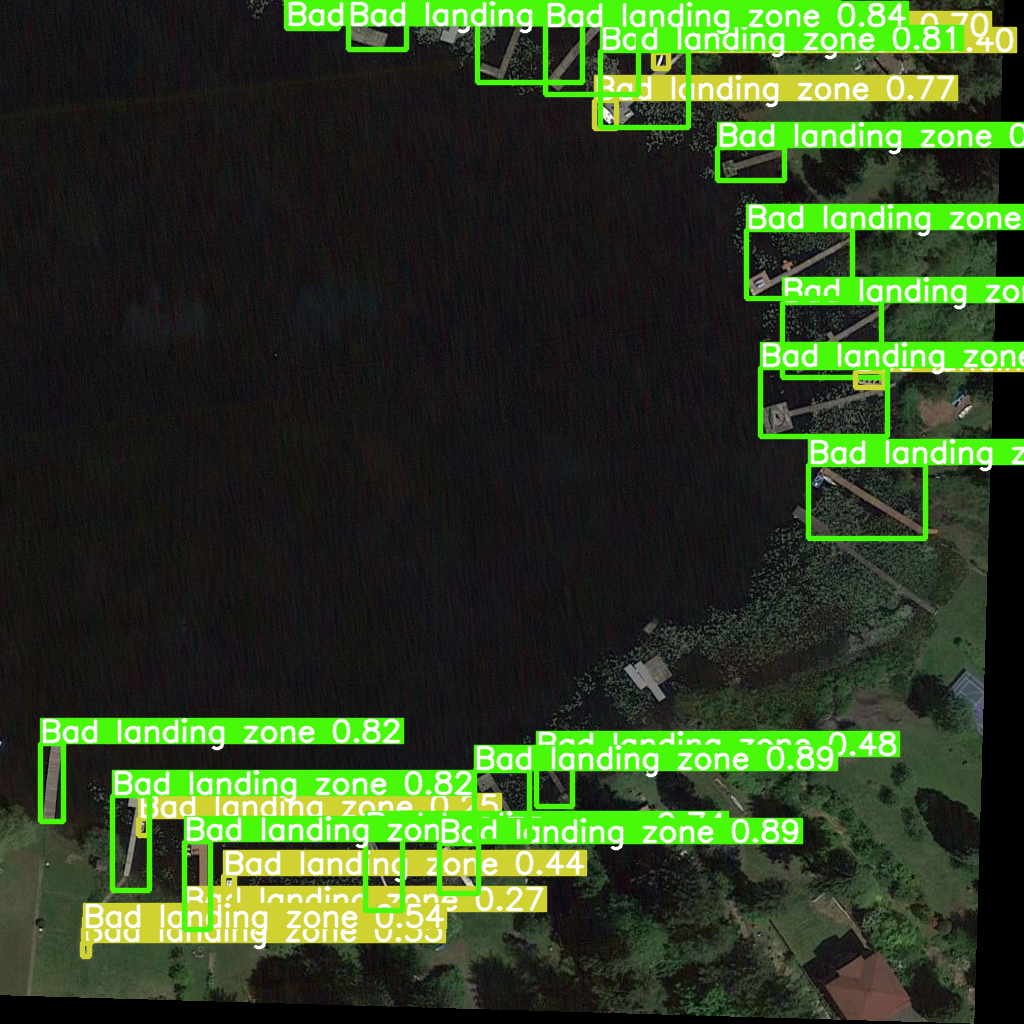

Supplement: Supplementary file 1 [file sensors-22-00464-s001.zip › Images/YOLOv5/Picture3.png]

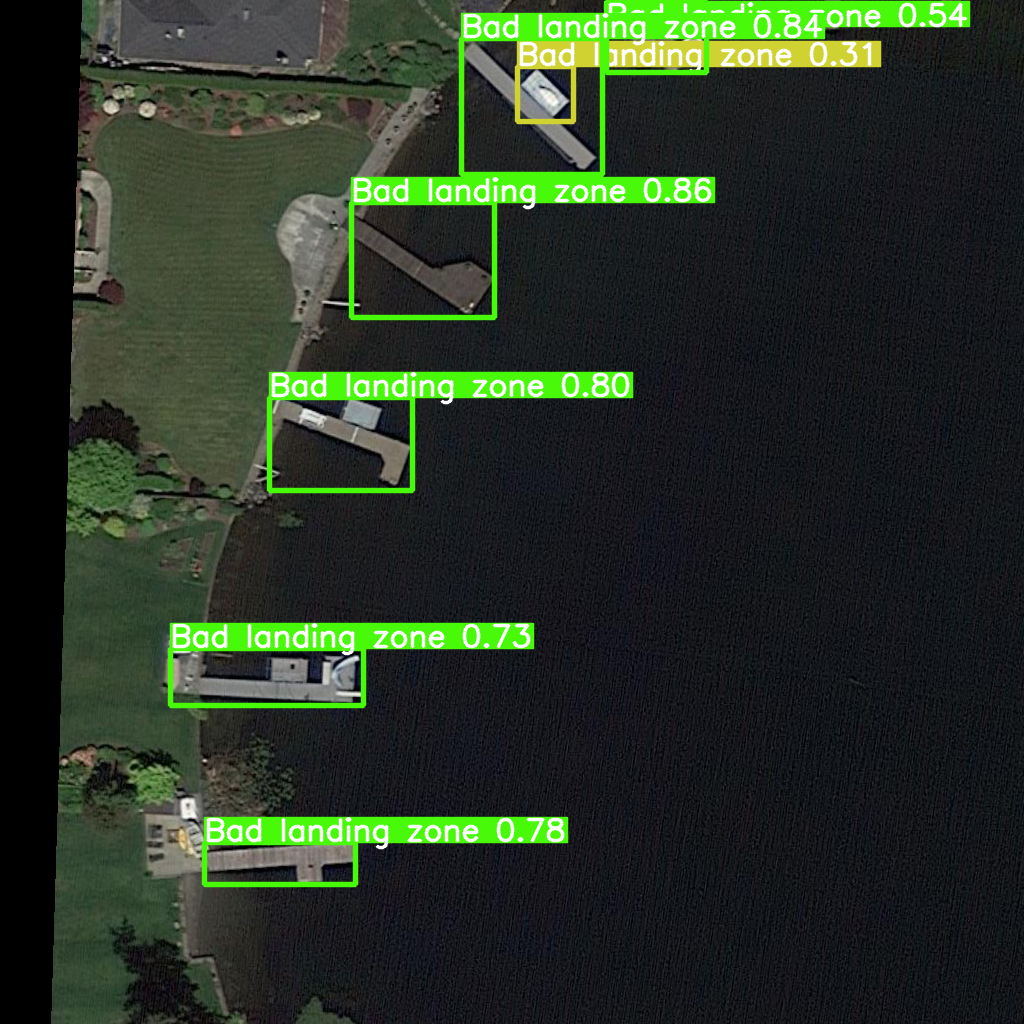

Supplement: Supplementary file 1 [file sensors-22-00464-s001.zip › Images/YOLOv5/Picture4.png]

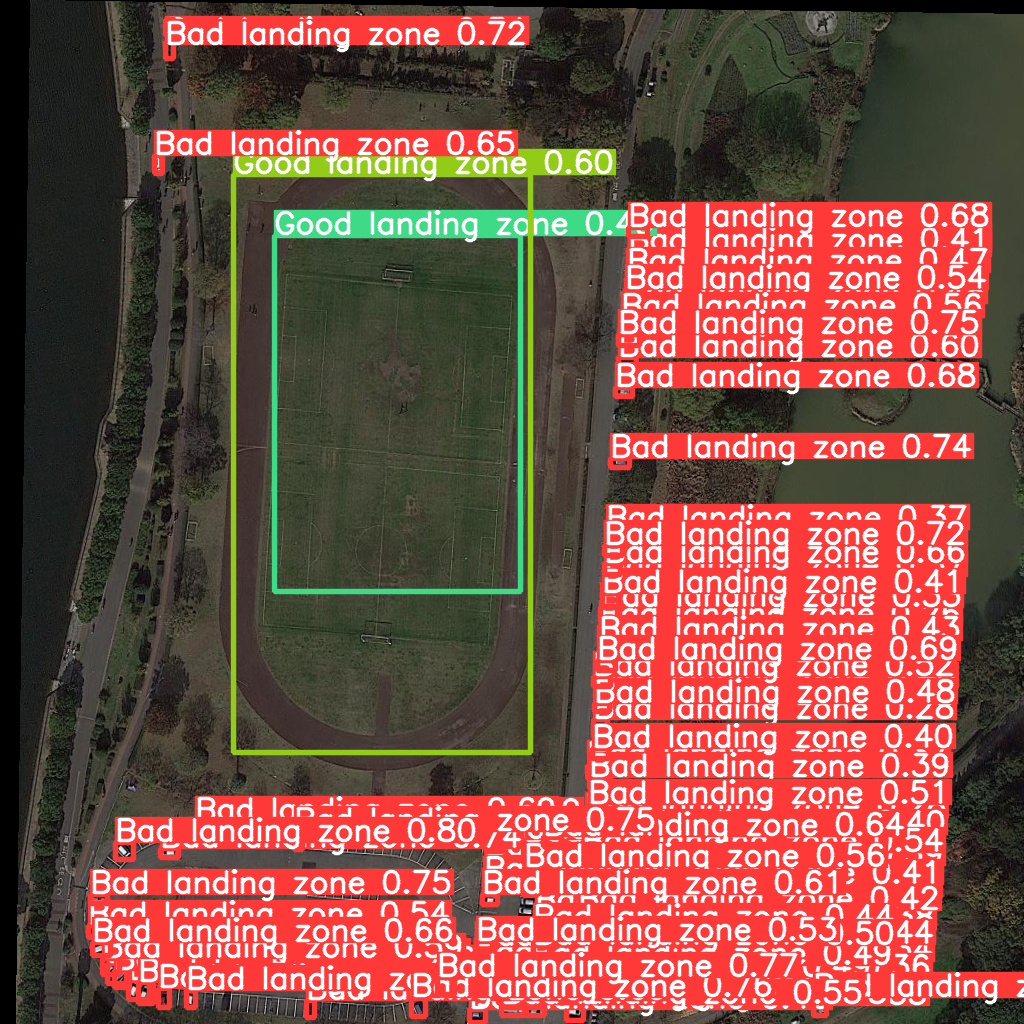

Supplement: Supplementary file 1 [file sensors-22-00464-s001.zip › Images/YOLOv5/Picture5.png]

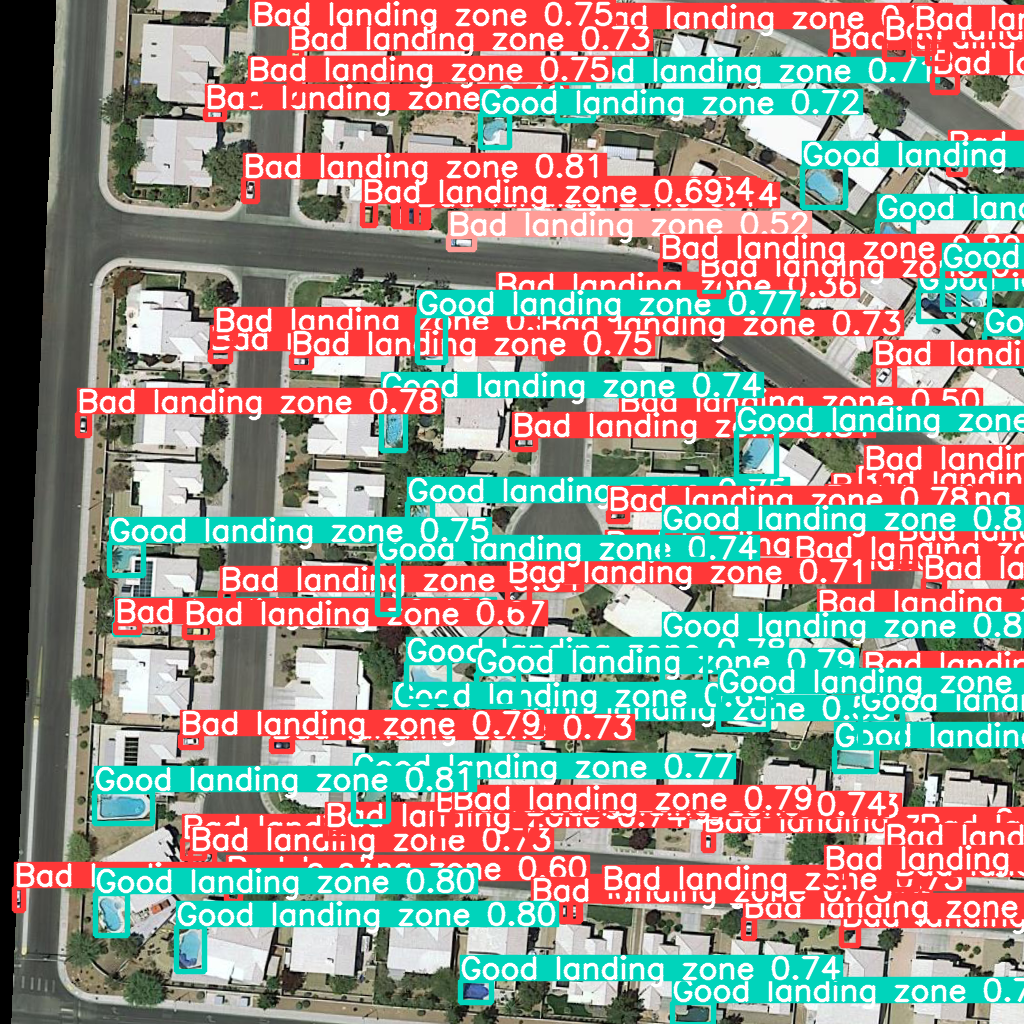

Supplement: Supplementary file 1 [file sensors-22-00464-s001.zip › Images/YOLOv5/Picture6.png]

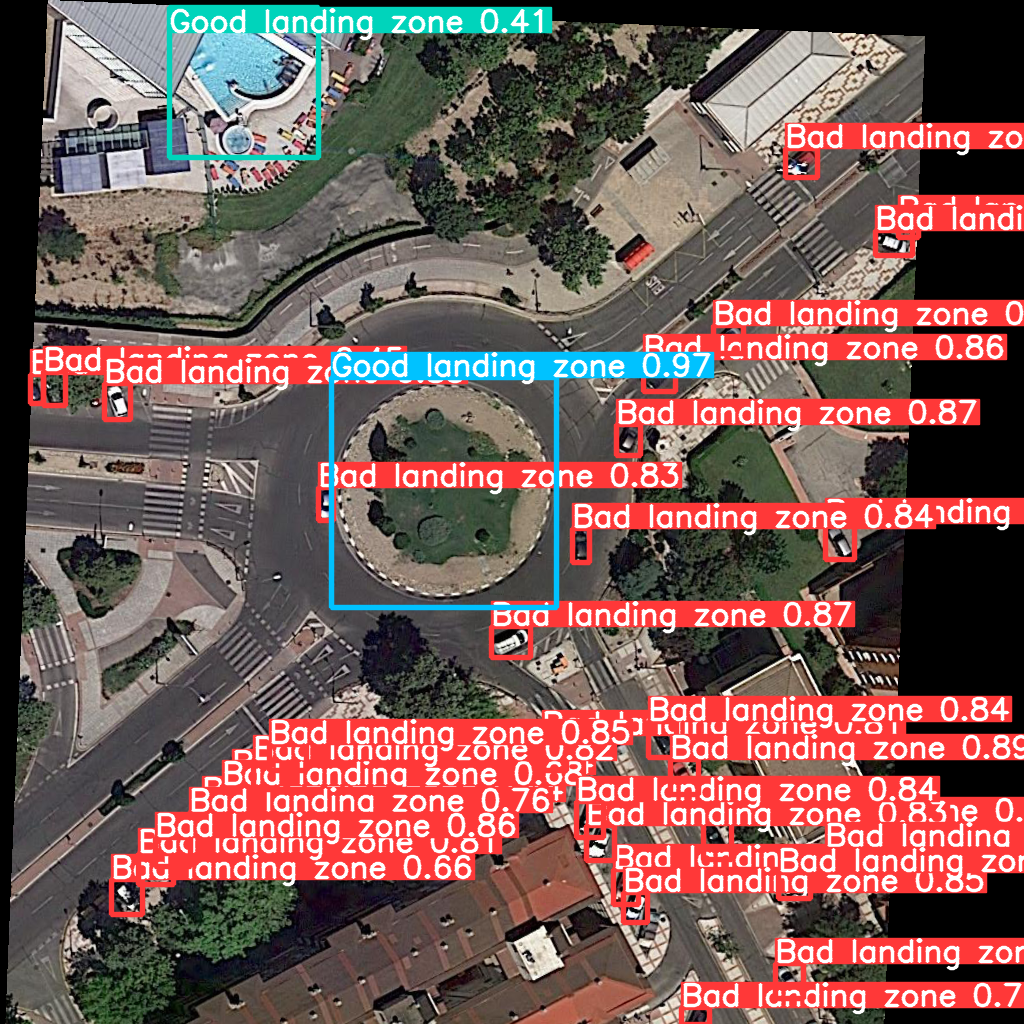

Supplement: Supplementary file 1 [file sensors-22-00464-s001.zip › Images/YOLOv5/Picture7.png]

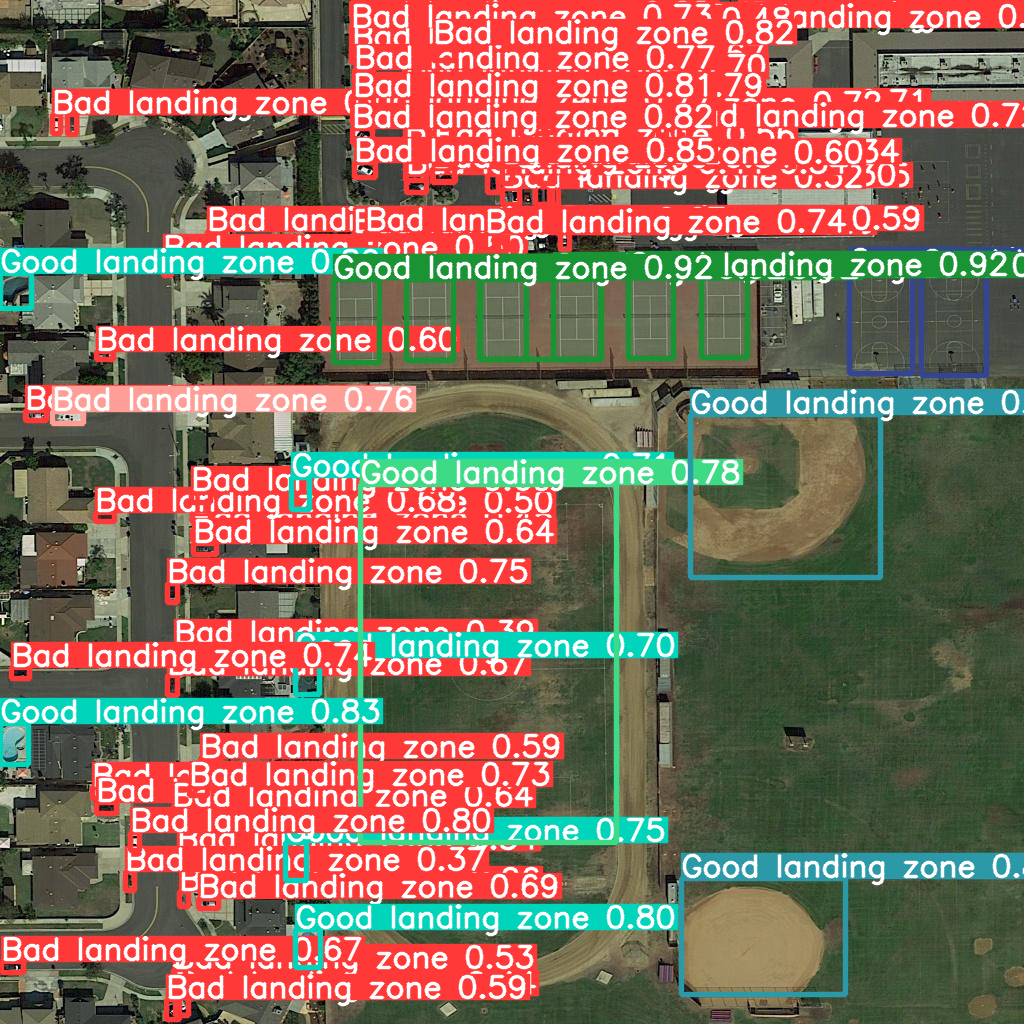

Supplement: Supplementary file 1 [file sensors-22-00464-s001.zip › Images/YOLOv5/Picture8.png]

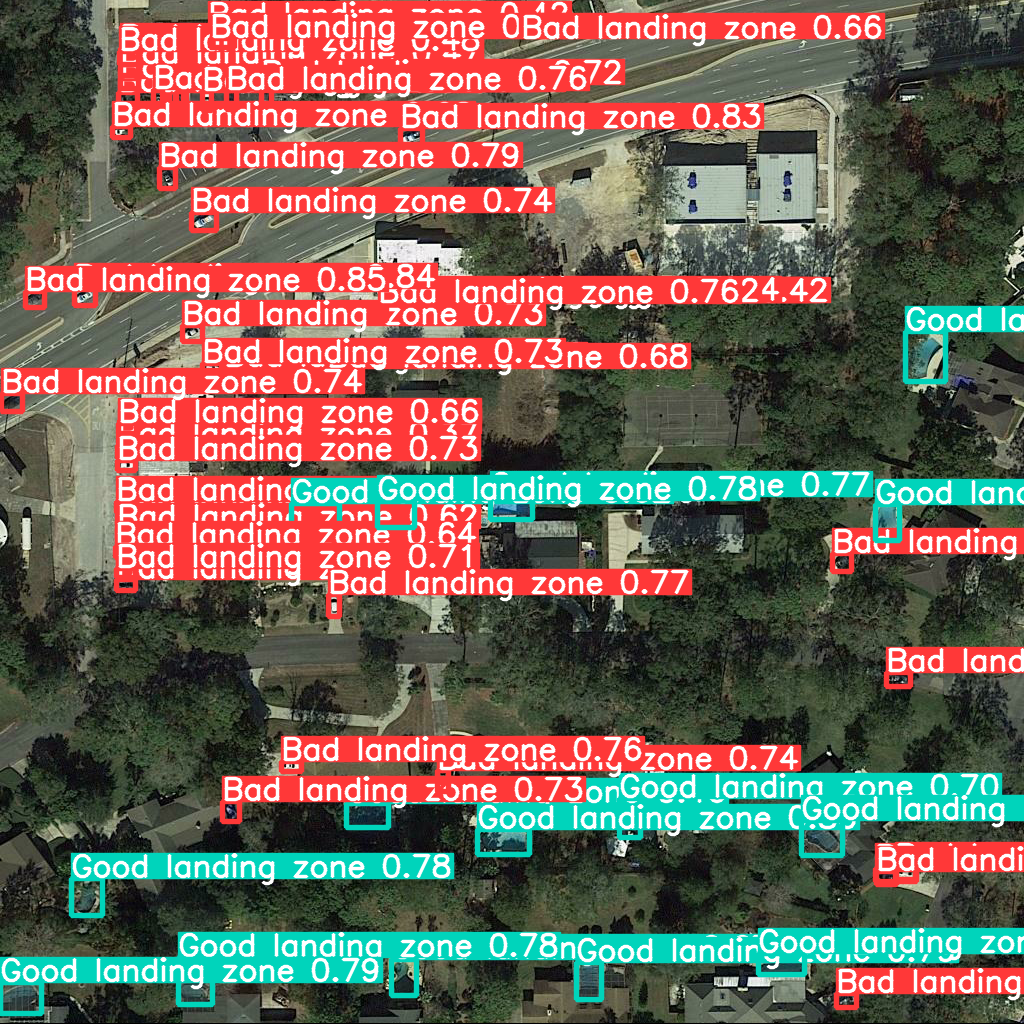

Supplement: Supplementary file 1 [file sensors-22-00464-s001.zip › Images/YOLOv5/Picture9.png]
